# The Geographical Variation of Colour Change in the Arabian Killifish (*Aphaniops dispar* Sensu Lato) From Freshwater and Marine Ecosystems

**DOI:** 10.1002/ece3.73005

**Published:** 2026-02-17

**Authors:** Ateah Alfakih, Nicola J. Nadeau, Penelope J. Watt

**Affiliations:** ^1^ Department of Biology, Faculty of Science Albaha University Albaha Saudi Arabia; ^2^ Ecology and Evolutionary Biology, School of Biosciences University of Sheffield Sheffield UK

**Keywords:** Arabian killifish, background matching, camouflage, colour change, geographical variation, plasticity

## Abstract

Widely distributed animals may exhibit substantial plastic physiological, morphological and behavioural responses to environmental changes. One such extraordinary form of plasticity is colour change, which varies substantially among most taxonomic groups. Most studies on colour change have focused on a single population, which has left a gap in our knowledge of colour change variation between populations. Examining colour change variation across populations of a taxon can shed light on how it evolves. Colour plasticity is influenced by spatial and temporal contextual variables, including visual habitat heterogeneity, predator diversity and their interaction. Using the Arabian killifish (*Aphaniops dispar* sensu lato) as a model, we examined the geographical variation of colour change in two freshwater and two marine populations from the west of Saudi Arabia, separated by vicariant and distance barriers and that differed in habitat visual characteristics and potentially in predator diversity. We used digital photography and visual modelling to investigate the ability of individuals in each of the populations to change colour when presented with achromatic (black and white) and chromatic (beige, brown and green) backgrounds. When presented with a black background, we found that individuals in all populations were capable of becoming darker at varying rates, but in a similar manner. Becoming lighter was more challenging, with individuals from all populations changing less on white than on black backgrounds and with one freshwater population exhibiting almost no response. However, there were complex interpopulation differences in chromatic responses, with marine populations exhibiting generally greater responses to the given backgrounds than freshwater populations. Here, we discuss the evolutionary and ecological factors possibly underlying these variations and their repercussions.

## Graphical abstract

Animals that live in a wide range of environments possess plastic traits such as colour change, but most research has only looked at this within single populations. In this study, we tested four populations of the Arabian killifish from marine and freshwater ecosystems, examining how well they change colour against different backgrounds. The study found differences in the ability to change colour between populations, with marine populations generally showing more colour‐changing responses than freshwater ones; the study highlights how environmental and evolutionary pressures may shape colour change among populations.

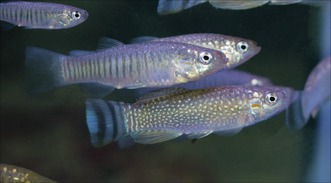

## 1 Introduction

Animals that are widely distributed and inhabit diverse habitats with different biotic and abiotic compositions may vary in their physiological (Staurnes et al. 1992; Cooper et al. 2018), morphological (Endler 1978; Macedonia et al. 2003) and behavioural characteristics (Foster 1999; Foster and Endler 1999). Animals' behaviours are often associated with their colouration, and colouration has been shown to play a crucial part in providing vital biological functions (Endler 1978; Stevens and Merilaita 2011), including signalling (Poulton 1890; Endler 1978; Hutton et al. 2015; Dollion et al. 2020), thermoregulation (Smith, Cadena, Endler, Kearney, et al. 2016; Smith, Cadena, Endler, Porter, et al. 2016), ultraviolet screening (Garcia et al. 2004) and crypsis (Cott 1940; Stuart‐Fox and Moussalli 2011; Duarte et al. 2017). Rather than being a fixed trait, animal colouration can be evolutionarily labile, subject to many spatiotemporal influential factors (Caro et al. 2016), and it can vary within and between populations of the same species (Greenwood et al. 2011, 2012; Cullen et al. 2022). Furthermore, the colouration of animals in disconnected populations may differ in response to environmental factors (McPhail 1969; Endler 1983; Magurran 2005). The most impressive example of this can be found in multiple pigmented epigean (surface‐dwelling) cavefish species and their depigmented hypogean (subterranean) counterparts, which have lost their pigment owing to the lack of functionality (Dowling et al. 2002; Romero and Green 2005).

In addition to evolutionary change, many animals can maximise the benefit of their colouration by plastically changing it in response to their habitat, such as its visual properties (Ramachandran et al. 1996; Barbosa et al. 2008; Smithers et al. 2017; Green et al. 2019), temperature (Smith, Cadena, Endler, Kearney, et al. 2016; Smith, Cadena, Endler, Porter, et al. 2016), the presence of heterospecifics (Kodric‐Brown 1983, 1998) or conspecifics (Ligon 2014), and ambient light (Vroonen et al. 2012). Colour change is categorised broadly into two subtypes (Bagnara and Hadley 1973): morphological colour change, which is slow (days to months) and involves an increase or decrease in pigment, and physiological colour change, which is rapid (seconds to minutes) and involves a movement of pigment. The capacity to change colour can vary substantially among most groups of colour‐changing animals (Stuart‐Fox and Moussalli 2009), although this has been rarely examined (Cadena et al. 2017), in comparison to geographical variations in other behaviours. It is evident that colour change varies among closely related species. For example, investigations into 21 groups of dwarf chameleons (*Bradypodion* spp.) revealed differences in the facultative response (i.e., the ability to fine‐tune the response to multiple stimuli) of colour change to bird and snake predators, with some lineages responding differently and others similarly (Stuart‐Fox and Moussalli 2009). In addition, recent research on two sympatric species of scorpionfishes, *Scorpaena* spp., showed that black scorpionfish ( *S. porcus* ) aggregated 50% of their dispersed pigments faster than the Madeira rockfish ( *S. maderensis* ) when placed on white substrates after being acclimated on black backgrounds (John et al. 2023).

Although studying differences in colour change between closely related species can provide valuable insight into the evolution of colour change (Clarke and Schluter 2011), it is a clear knowledge gap (Stuart‐Fox and Moussalli 2009; Hutton et al. 2015; Duarte et al. 2017), and it remains difficult to infer how these variations have arisen among species. Examining the variation in colour change among populations of a specific taxon can, therefore, cast considerable light on the evolution of colour change. While most studies focused on a single population, a search of the literature revealed several lines of evidence that colour change varies between disconnected populations in terrestrial (Stuart‐Fox et al. 2006; Stuart‐Fox and Moussalli 2009; Munguia et al. 2013; Choi and Jang 2014; Batabyal and Thaker 2017; Cadena et al. 2017; Spadavecchia et al. 2022; Whiting et al. 2022) and in aquatic environments (Whiteley et al. 2009, 2011; Westley et al. 2013; Pinto et al. 2020). Differences in the ability to colour change do not appear to be attributed to a single factor or combination of factors applicable to all species, indicating that the processes that explain the evolution of population variation in colour change vary between and within species and remain inadequately understood.

Environment‐specific requirements, such as physiological (Alfakih et al. 2022), sociosexual, thermoregulatory and camouflage, as well as their interactions, may drive variations in colour change (Stuart‐Fox and Moussalli 2009). Generally, if some populations are subject to stronger natural selection for camouflage than others, it is anticipated that there will be differences in their capacity to change colour (Stuart‐Fox et al. 2006). Thus, spatial and temporal environmental differences play a significant role in shaping the evolution of plasticity, including colour change (Doughty and Reznick 2004). It is broadly predicted that both among and within habitats, the increase in variation of visual properties is associated with an increase in the capacity to change colour (Cadena et al. 2017), though this is not always the case. For instance, four populations of brown trout ( *Salmo trutta* ) were found to differ in the magnitude of their pigmentation change despite the similarity of their environments (Westley et al. 2013), and comparable findings were observed in dwarf chameleons ( *Bradypodion transvaalense* ) (Stuart‐Fox et al. 2006). The majority of animals, however, reside in ecosystems that have several predators in their microhabitats (Stuart‐Fox and Moussalli 2009), which possess different visual capacities, and differ in their reliance on visual versus other sensory cues. Thus, prey species may evolve increased colour‐based defensive tactics (Caro et al. 2016), and selection for increased colour change capacity may accord with the increase in visually guided predator diversity (Whiteley et al. 2011) or abundance (Stuart‐Fox et al. 2006; Stuart‐Fox and Moussalli 2009).

Nonetheless, the effects of predator diversity on colour change per se have not been adequately addressed and are only marginally acknowledged. Stuart‐Fox and Moussalli (2009) provided evidence that suggests the loss of facultative colour change in *Bradypodion* chameleons may be an adaptation to geographically variable local predation regimes. More recently, this suggestion has been reinforced by findings showing that reduced predation pressure influenced the extent of colour change in the invasive Jackson's chameleon ( *Trioceros jacksonii xantholophus* ) in Hawaii (Whiting et al. 2022). Predation and visual habitats are inseparable factors that affect colour change. Antipredator responses vary spatially depending on predator exposure, leading to population‐specific strategies and colouration‐based behaviours (e.g., fish: Magurran et al. 1993).

**FIGURE 1:**
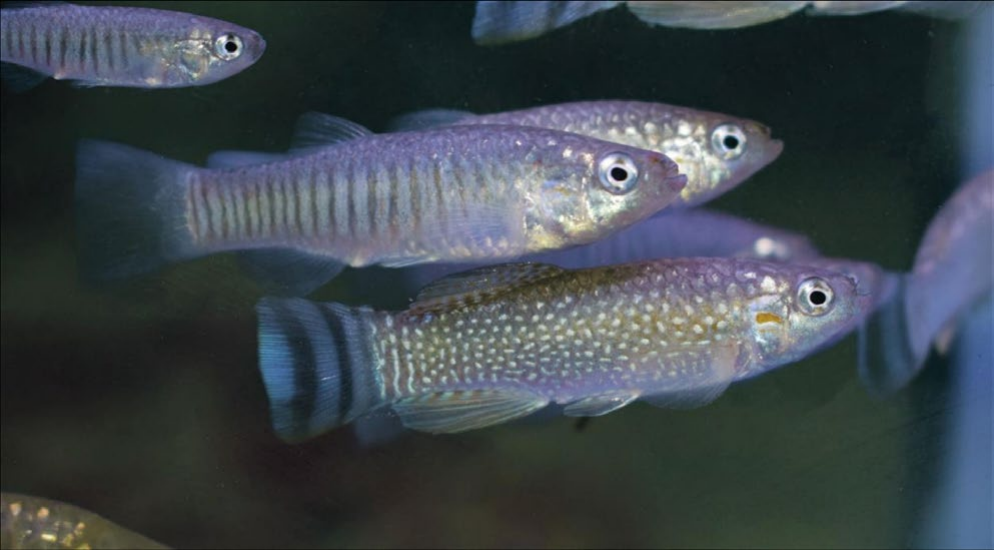
A male (bottom) and females (top) of the Arabian killifish *A. dispar* from Wadi Al Arj, Saudi Arabia.

Euryhaline fish species (which tolerate a broad range of salinity) are highly adaptable to various ecological systems and may potentially become landlocked (Schultz and McCormic 2013). This adaptability makes them valuable models for studying geographical resilience in phenotypic traits, such as colour change. Here, we examine the geographical differences in achromatic and chromatic colour change between populations of the Arabian killifish (*Aphaniops dispar* sensu lato) (Figure 1) from the Red Sea basin in Saudi Arabia. Natural populations of the Arabian killifish have the capacity to change colour (personal observations), but this ability in the genus *Aphaniops* has not been officially documented. This sexually dimorphic species is euryhaline and eurythermal, meaning that it can tolerate a broad spectrum of water salinity and temperature (Haas 1982; Krupp 1983). Hence, it has a wide geographical range and thrives across multiple ecosystems, including hypersaline marine and freshwater ecosystems, with some populations believed to be landlocked (Krupp 1983). Here, we present the first evidence for colour plasticity in members of the *Aphaniops dispar* complex, and variation in colour change among different ecosystems, comprising two freshwater and two marine populations that are geographically separated by distance and land barriers.

The Arabian killifish generally lives in shallow water (Bonzi et al. 2021), whereas in marine systems, it occupies shallow lagoons, mangrove forests (Walton et al. 2021) and coral reefs (Freyhof et al. 2020). The lagoon locations on a large scale are consistently homogeneous across many sites on the Saudi Arabian and Yemeni Red Sea coasts (Figure S1; Krupp et al. 2006; Rasul 2015; Scott et al. 2022). Lagoons mostly consist of sand and mud, light yellowish to dark brown in colour, with the latter likely derived from freshwater wadis (ephemeral streams) during floods (Rasul 2015; Scott et al. 2022). In comparison, wadis are more visually heterogeneous, consisting of aquatic plants, eutrophicated patches, rocks and gravel pools (Figure S2; García et al. 2015). The Arabian killifish is susceptible to predation (Figure S3) from birds in both ecosystems and from snakes in freshwater environments (Egan 2007; Freyhof et al. 2020). In freshwater environments, the most diverse fauna is generally found in the southernmost regions of Saudi Arabia (Gasperetti 1988; Egan 2007; Boland and Alsuhaibany 2020). In marine habitats, fish are key predators of the killifish (Zajonz et al. 2002; Lieske and Myers 2004; Coad 2010), such as the crescent grunter ( *Terapon jarbua* ), which is known to hunt *Aphaniops* species (Llewellyn‐Smith 2011), but no piscivorous fish are present in freshwater habitats. These profound differences in the visual environments and potentially in patterns of predator diversity could select for differences in colouration or colour change ability between marine and freshwater populations.

This research aimed to quantitatively measure the ability of the Arabian killifish to change colour and to examine whether there is any geographical variation in their ability to change achromatically (luminance: the perceived brightness by the visual model as detailed below) in response to black and white backgrounds and chromatically (hue: the actual colours, and saturation: the intensity of colour) in response to beige, brown and green backgrounds. Here, we hypothesise that colour change will vary according to the general visual characteristics of the environments, predicting that populations occupying more visually diverse environments will exhibit greater achromatic and chromatic colour‐changing ability in terms of both speed and magnitude.

## 2 Methods

### 2.1 Study Species and Collection Sites

**FIGURE 2:**
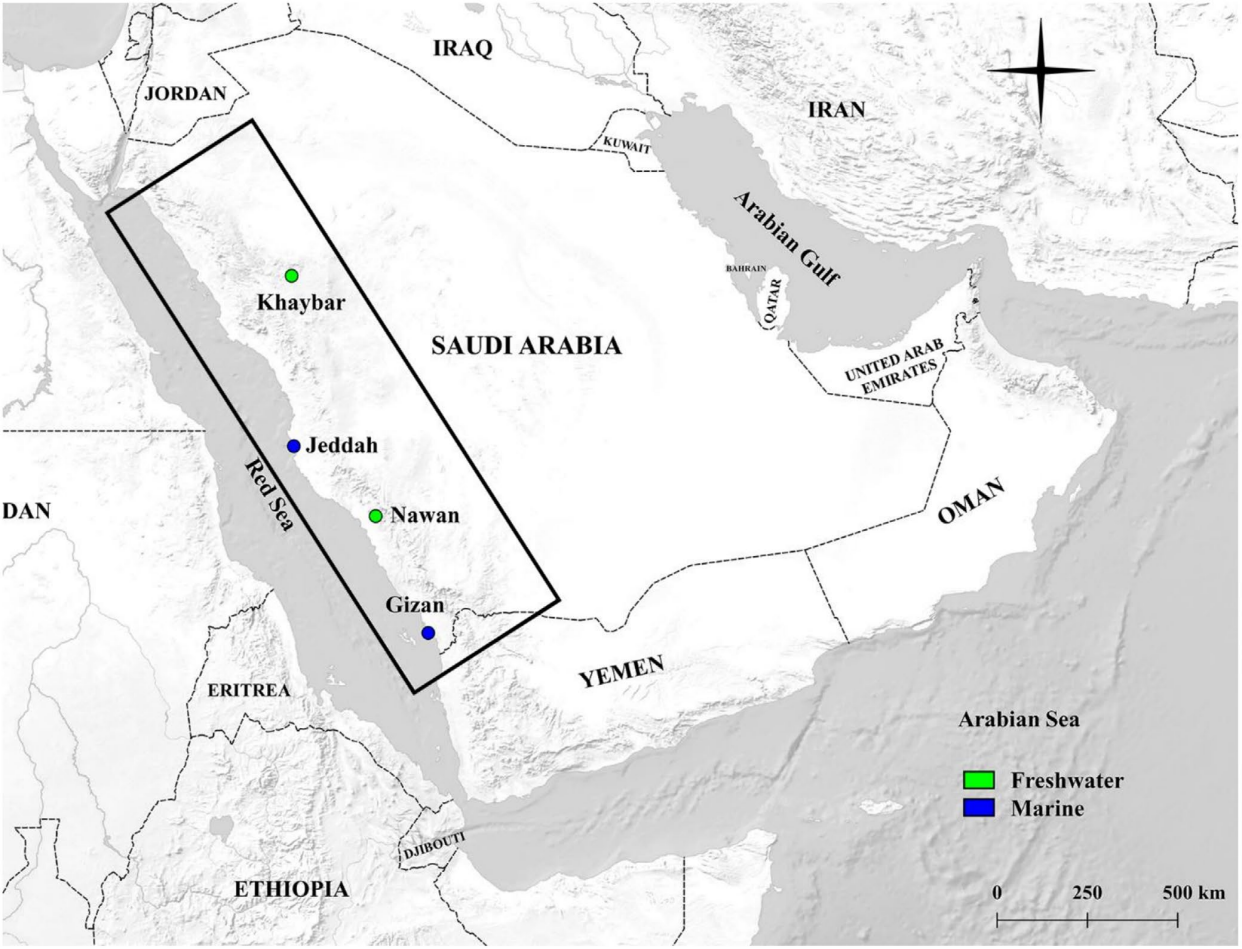
A map illustrating the locations of the four populations studied, including two marine populations represented by blue circles (Gizan and Jeddah), two freshwater populations represented by green circles (Khaybar and Nawan).

**FIGURE 3:**
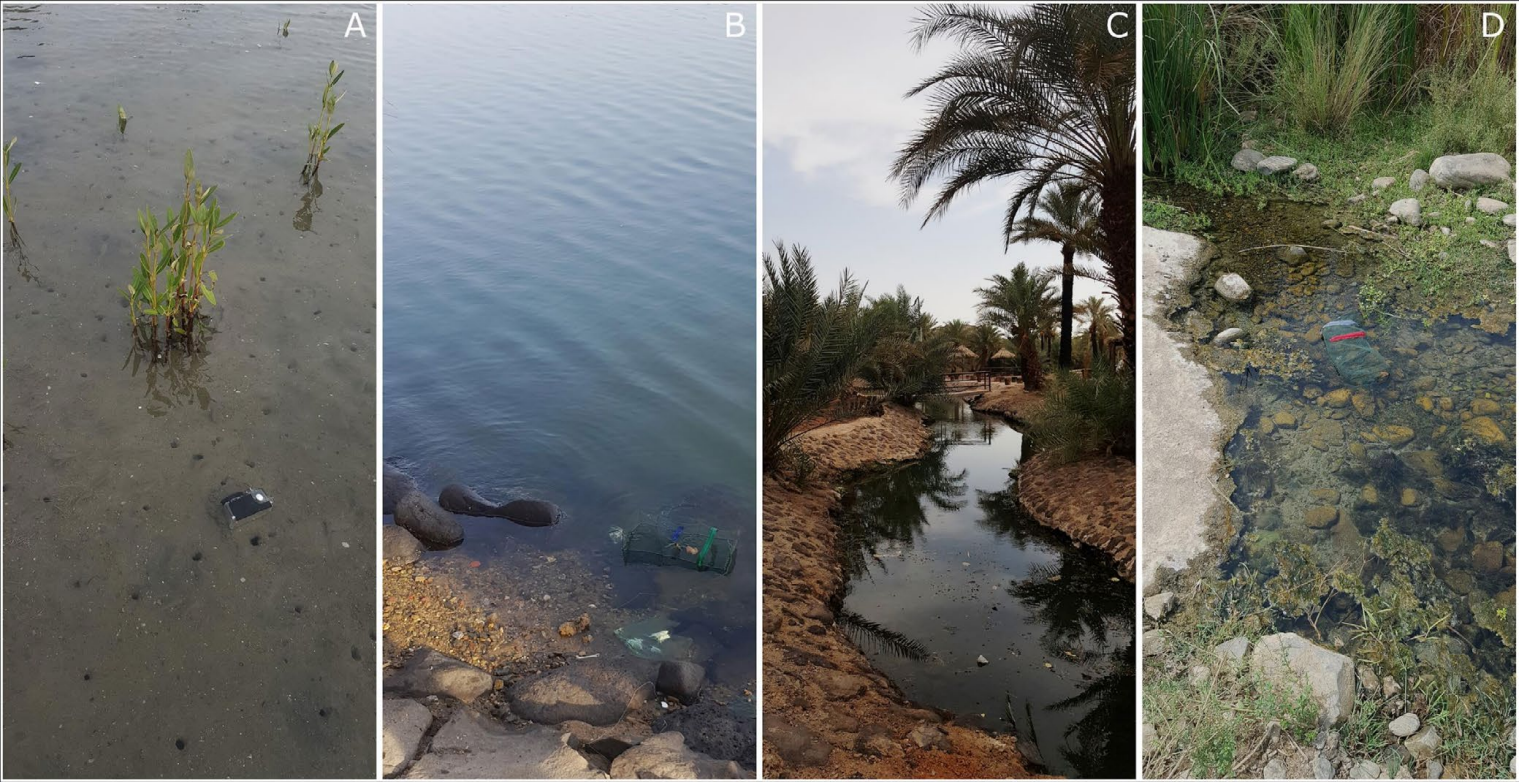
Some representative images of the visual habitats in the study areas showing the marine locations (A) Gizan and (B) Jeddah and the freshwater locations (C) Khaybar and (D) Nawan.

We collected mixed‐sex wild‐caught individuals of the Arabian killifish *A. dispar* sensu lato (Figure 1) opportunistically from four locations in the west of Saudi Arabia, including two marine populations: Gizan (16°50′07.9″N 42°34′34.4″ E) and Jeddah (21°29′24.9″N 39°10′58.9″ E) and two landlocked freshwater populations from internal wadis: Khaybar (25°44′38.6″N 39°15′19.3″ E) and Nawan (19°39′25.1″N 41°16′57.7″ E) (Figure 2). The sampling locations were chosen based on the ecosystem types (freshwater and marine) and the latitudinal distance between the populations to represent the extreme allopatry of this species, while also encompassing differences in associated fauna, including predators and competitors. In marine environments, the salinity was typically around 35 parts per thousand (ppt), whereas in freshwater streams, the salinity was nearly 0 ppt. The marine locations were predominantly homogeneous, with Gizan consisting of light brown sand, grey gravel with some seagrass and algae (Figure 3A) and Jeddah primarily composed of large dark rocks with crevices and some greyish gravelled and light patches and some algae (Figure 3B). The substrates at the northern freshwater location Khaybar were highly dark brown with some green biofilms (Figure 3C), where the population is located in small springs surrounded by farms and basaltic volcanic rocks. The southern freshwater location Nawan, which was more visually heterogeneous than the other study locations, consisted of brown to dark grey sand, brown and green algae, reed plants and gravel with varying brightness (Figure 3D). Based on ongoing genetic work (unpublished), the Gizan population appears to form a distinct molecular operational taxonomic unit (MOTU), whereas populations from Jeddah, Khaybar and Nawan cluster within a second MOTU. However, these genetic relationships have not yet been formally confirmed and are not tested in the present study. Accordingly, populations are treated here as geographically and ecologically distinct units for the purpose of examining colour plasticity.

### 2.2 Fish Collection

Four hundred fish in total (*n* = 100 fish per site) were used in the study, which were collected using cuboid‐shaped nylon traps (L 54 × W 25 × H 25 cm) to avoid any harm or damage to them. This was done by placing bread bait inside the traps and submerging them in shallow water at random places for a few minutes. Fish were gently removed from the traps and placed in 12 L plastic containers containing water from their collection site to reduce stress and portable air pumps were used to provide oxygen. Fish from each population were housed indoors near the collection area in two tanks for each experiment (L 50 × W 30 × H 40 cm: ~20 fish/tank). The salinity was maintained for each population by housing individuals from the freshwater population directly in freshwater, while the marine fish were placed in saline water at ~35 ppt by mixing fresh water with Red Sea salt mix (Red Sea). Since the Arabian killifish is eurythermal (Haas 1982; Coad 2010), the temperature was maintained at ~28°C using tank heaters, which is within the recorded water temperature of both ecosystem types (Bonzi et al. 2021; Schunter et al. 2021). Each tank was kept under a 12:12 h light: dark cycle and supplied with a tank pump with a filter to circulate and oxygenate the water, and fish were fed twice daily with commercial flake food (Siso Bio‐tech CO. LTD). Fish were acclimated to the husbandry tanks under the above conditions for at least 24 h before being used in the experiments.

### 2.3 Ethical Note

The study was carried out with permission from the Saudi Wildlife Authority (2020) before it commenced. The collection sites were open to the public, and no additional licences or permits were required to access them except for springs in Khaybar, for which a separate access permit was acquired from the Royal Commission for AlUla (2022). The Arabian killifish is neither threatened nor protected (Freyhof et al. 2020). Moreover, the study was not invasive and complied with the Saudi Law of Ethics of Research on Living Things (R. D. No. M/59: 24 August 2010) and the EU animal welfare rules (Directive 2010/63/EU) recommended by ASAB (2020). All the fish were returned to their original site after completing the experiments.

## 3 Background Creation and Experimental Design

### 3.1 Achromatic Backgrounds

The aim of the first experiment was to determine whether individuals from the freshwater and marine populations were capable of altering their luminance when exposed to achromatic (black and white) backgrounds. Each background was made of waterproof paper (Xerox Premium NeverTear Paper; Antalis, London, UK). We used the Xerox white paper for the white backgrounds and measured its mean reflectance (see Image analysis for reflectance measurements), which was 85.5%. Black backgrounds were generated in Inkscape version 0.92.3 (https://inkscape.org) by specifying the RGB values to 0:0:0 (Smithers et al. 2017). The black backgrounds were printed with an HP Colour LaserJet M254dw printer at 600 dpi (Hewlett‐Packard, USA), and the mean reflectance of the paper was 5.9%. To acclimate the fish prior to the experiments and to ensure that they all had the same starting point, midpoint grey backgrounds with an intermediate reflectance relative to the average reflectance of the black and white backgrounds were created using a method similar to that of Kang et al. (2016) and Smithers et al. (2017). We created 100 greyscale gradients in Adobe Photoshop 2019 (Adobe Systems), resulting in a grid of different greyscale blocks with RGB values ranging from (black) 0 to (white) 255 (Figure S4). The grid was printed and photographed (see Photography methods below), and the reflectance of each grid block was measured. The closest midpoint grey colour between the black and white papers was selected to generate another grid. This process was repeated until we obtained an intermediate grey substrate which reflected 45.7% with an RGB value of 151 (Figure S4).

### 3.2 Chromatic Backgrounds

The chromatic backgrounds were designed primarily using the previously described approach. Rather than indiscriminately selecting colours, the objective was to produce broad representations of the sort of natural backgrounds the fish would experience and have adapted to in the wild. This approach prioritised approximating the chromatic backgrounds rather than perfectly replicating them, as the study focused on colour change rather than habitat matching. Therefore, prior observations were made during this study to assess the broad visual characteristics of different marine and freshwater locations in the west of Saudi Arabia to cover as many potential natural colours as possible (Figures S1, S2). We subjectively divided the colours of the observed habitats into one common background and two opposing backgrounds. The colour green was selected for the common substrate as it was similar to the algal substrates seen usually in the freshwater and marine ecosystems. To reduce complexity, we utilised colours of the sand for the opposing backgrounds, which varied slightly in both habitats, being primarily brown in freshwater streams and beige in coastal areas (Figures S1, S2); the light sand colour (beige) seems to be a consistent feature of the Saudi Arabian Red Sea coast (Scott et al. 2022).

We photographed wet algal and sand substrates from the marine and freshwater locations from a height (~1.5 m) in the visible human spectrum because water absorbs a substantial portion of light, and light penetration varies with water depth, affecting the colours and reflectance of backgrounds. The images were captured in the shade to eliminate overexposure, using a Nikon D3300 equipped with an 18–55 mm Nikon lens in raw format and had a Spectralon 99% reflectance standard. The white balance of these photographs was then corrected in Camera Raw (Adobe Systems) using the reflectance standard. The average values of the RGB channels of the substrates were then measured in Photoshop and input to Inkscape to print the final colours (Figure S5). In this experiment, we intended to examine the changes in the fish's actual colours (hue and saturation) by placing individuals from the four locations on chromatic backgrounds. The perceived differences in brightness between the created starting backgrounds (i.e., the grey acclimating backgrounds) and the printed coloured backgrounds were eliminated. This was done by measuring the average reflectance of the printed coloured backgrounds and then selecting the corresponding grey starting points with a similar reflectance to the printed coloured backgrounds, in the same way as described for the achromatic backgrounds above (Figure S5; Table S1).

### 3.3 Experimental Procedure

For each population, we used 20 different and randomly selected mixed‐sex fish for each type of the achromatic background: black and white, and the chromatic background: beige, brown and green. In each experiment, each fish was placed and kept for 10 min in an oxygen‐supplied tray (L 12 × W 12 × H 10 cm) containing the starting grey backgrounds under constant photography conditions (see below). This was to ensure that all the individuals had the same starting point, allowing them to acclimatise and diminish any colour changes induced by stress (Stevens, Lown, and Denton 2014; Smithers et al. 2017). After being acclimated for 10 min, the individuals were swiftly transferred to the photography containers with the test backgrounds (L 7 × W 7 × H 7 cm) and immediately photographed (minute 0), and at minutes 1, 8 and 15. The photography containers were filled with oxygenated water that was salinity‐matched to their original locations (0 ppt for freshwater and 35 ppt for marine) and had a temperature of ~28°C that matched the husbandry conditions. Acclimating and photography containers were washed with water after each experiment to avoid olfactory cues being transferred to subsequent test individuals, and tested fish were returned to the acclimating containers for 10 min and then to another husbandry tank and provided with food. Transferring fish to and from the photography containers was performed using a small, transparent plastic tray immersed in water; this method proved efficient in reducing stress because fish did not exhibit flight (escape) response to this handling.

### 3.4 Photography

We used digital photography as a non‐invasive approach to quantify colour and colour change. Photographs of the fish were captured in a photography room with a Nikon D7000 digital camera that had been converted to capture images in the visible and ultraviolet (UV) spectra (Advanced Camera Services, Norfolk, U.K.). The camera was equipped with a Nikon 105 mm f/2.8G Micro‐Nikkor lens and placed approximately 95 cm above the photography containers utilising a tripod. Due to the high mobility of the fish, it was challenging to take photographs in the UV range, so only images in the visible spectrum 420–680 nm were taken using a Baader UV/IR‐Cut/L filter (Baader Planetarium, Mammendorf, Germany). All photographs were captured in raw format (.NEF) with fixed ISO (400) and aperture (F3) under EyeColor Arc MT70D lamp (Iwasaki Electric Co. LTD). The shutter speed was manually adjusted depending on the background colour but maintained across the study for all of the populations (i.e., each background colour had a fixed shutter speed for all the 4 studied populations). The light was diffused using a custom‐built tent made with PVC pipes 110 × 80 × 125 cm and covered with white PEVA covers from the sides as well as with a black‐matte foam board over the tent to prevent specular reflection. Each image had a known scale bar of 3 cm along with 4.88% and 41.73% reflectance standards (ColorChecker Passport MSCCPP‐B: X‐Rite) for all background types except for the white background, which had 41.73% and 98.24% reflectance standards. These standards were chosen to fit within the expected reflectance values of the fish and backgrounds. The photography settings were maintained throughout the study, and photographs were captured from a uniform distance and angle across the study.

### 3.5 Image Analysis

The aim of this study was to examine the geographical variations in *A. dispar'*s ability for achromatic change (luminance) and chromatic change (hue and saturation), in addition to see if the changes affected luminance and colour camouflage. Images were analysed with ImageJ software (Schneider et al. 2012) using the Multispectral Image Calibration and Analysis Toolbox (MICA) (Troscianko and Stevens 2015). This tool linearised and standardised the raw nonlinear images of the fish and their backgrounds to calibrated linear multispectral images with regard to the 4.88%, 41.73% and 98.24% grey standards, which encompassed reflectance information of the visible RGB channels. The linearisation of the photographs was used to control for any fluctuations in light conditions to obtain objective measurements (Stevens et al. 2007; Troscianko and Stevens 2015). The dorsal view of each individual from the snout tip to the base of the caudal fin (excluding the pectoral fins and the dorsal fin when it was extended over the body) was chosen as a region of interest (ROI) for luminance, colour and camouflage quantification. We randomly selected ~1 cm^2^ of the photographed test backgrounds for each image as a ROI at minute 0. The collected fish had an average length of 3.5 cm ± 0.67 (standard deviation), measured from the tip of the snout to the base of the caudal fin using ImageJ.

In order to address the biological significance of the changes in the colouration of the Arabian killifish (luminance, luminance camouflage and colour camouflage), they were analysed from a predator visual model. Despite the lack of data concerning the key predators of *A. dispar* (but see examples in Coad 2010 for fish and Freyhof et al. 2020 for birds) and their visual systems, this fish and cyprinodontiformes in general are likely to be important prey to birds in all ecosystems (Figure S3; Silva et al. 2019). Thus, the calibrated photos were mapped from the camera spectral sensitivity into the trichromatic version of the peafowl's ( *Pavo cristatus* ) spectral sensitivity (Hart 2002) in MICA (Troscianko and Stevens 2015), which has been frequently used as a model of avian vision (e.g., Kang et al. 2015; Barnett et al. 2018; Nokelainen et al. 2020). The mapping technique resulted in images with channel values corresponding to long‐wave (LW), medium‐wave (MW), short‐wave (SW) sensitive cones and double cone type (dbl).

The changes in luminance were directly analysed from the dbl catch values of the peafowl model. Camouflage due to changes in luminance and colour was analysed against the test backgrounds as perceived by the avian predator model and expressed as Just Noticeable Differences (JNDs). The achromatic camouflage was calculated via the modified version (Siddiqi et al. 2004) of Vorobyev and Osorio's (1998) receptor noise limited model (RNL). We used a Weber fraction of 0.2, as reported by Olsson et al. (2018). For the chromatic camouflage, we used a Weber fraction of 0.06 (Olsson et al. 2015) as a noise for the most abundant cone cell of the peafowl visual model, which is MW based on the relative abundance of the cone types of the peafowl (SW 1.9: MW 2.2: LW 2.1) (Hart 2002). Then we estimated the noise in the SW and LW cones using $e_{i}=\sqrt{\frac{\eta_{\mathrm{MW}}}{\eta_{i}}}\;e_{\mathrm{MW}}$ (Olsson et al. 2018; Supporting Information), where $e_{i}$ is the noise in the cone type i (SW or LW), $\eta_{\mathrm{MW}}$ is the relative density of the MW cone which is 1, $\eta_{i}$ is relative density of the cone type i (calculated as a ratio to the MW cone) and $e_{\mathrm{MW}}$is the Weber fraction 0.06 of the MW cone. The final noise in each of these photoreceptors was (SW = 0.0645; MW = 0.06; LW = 0.0614). Based on the discrimination model described above, the obtained digits of the luminance and colour JNDs specified whether the luminance and colour of two objects were perceptible to the receiver model. Generally, a JND value of < 1 indicates that the two stimuli are indistinguishable by the receiver vision, and values between 1 to 3 might mean the two stimuli can be detectable under good viewing conditions. Increment in JNDs over 3 shows that the two objects' colours become more distinguishable (Stevens, Lown, and Denton 2014).

In order to measure the saturation and hue of fish and backgrounds, we first standardised each of the LW, MW and SW catch values to remove the brightness differences (Stevens 2011). This was done by dividing each catch value by the sum of all catch values (e.g., $\mathrm{sLW}=\frac{\mathrm{LW}}{\mathrm{LW}+\mathrm{MW}+\mathrm{SW}}$) (Kelber et al. 2003; Stevens 2011). Then, these values were mapped into a trichromatic colour space using Cartesian coordinates where $x=\frac{1}{\sqrt{2\;}}\;\left(\mathrm{sLW}-\mathrm{sMW}\right)$ and $y=\frac{\sqrt{2}}{\sqrt{3}}\;\left(\mathrm{sSW}-\frac{\mathrm{sLW}+\mathrm{sMW}}{2}\right)$ (Kelber et al. 2003). Saturation was then calculated as the distance from the achromatic centre (Stevens 2011) in the trichromatic colour space, $\sqrt{x^{2}+y^{2}}$ in which higher values indicate that colours are more saturated. Hue is difficult to generate from colour space (Stevens 2011), thus, the hue was simply calculated as the difference between standardised middle to the longer‐shorter wavelengths, $\mathrm{sMW}-\frac{\mathrm{sLW}+\mathrm{sSW}}{2}$ similar to previous studies (e.g., Komdeur et al. 2005; Stevens, Lown, and Denton 2014; Smithers et al. 2018). A value of 0 from this calculation means that the subject measured is achromatic and has equal cone catch values across all three channels. According to the printed backgrounds, the beige, brown and green hues had average values of 0.005, 0.015 and 0.075, respectively. Therefore, we could determine whether changes in hue were directed to the backgrounds' hues.

### 3.6 Statistical Analyses

All statistical analyses were performed in R version 4.2.3 (R Core Team 2023). We performed linear mixed models via the “lme4” package (Bates et al. 2015), and significant differences were obtained from the package “lmerTest” utilising Satterthwaite's method (Kuznetsova et al. 2017). Each background treatment was analysed separately with the following colour metrics: luminance, hue, saturation, luminance JND and Colour JND as response variables; Colour JND was only analysed for the chromatic backgrounds (beige, brown and green). The test time (minutes 0, 1, 8 and 15), fish population and fish sex were treated as fixed categorical variables and fish length as a fixed numerical variable with the fish identities as random effects to account for the repeated measures.

Fish length and sex were treated as fixed covariates, and the final model syntax was as follows: Colour metric ~ TestTime * FishPopulation + FishLength + FishSex + (1|FishID). This simple formulation was used as the scope of this study is to investigate the interpopulation differences in colour change. Therefore, we avoided the complexity of over‐parameterisation and the inclusion of three‐ and four‐way interactions. To evaluate whether alternative model structures provided a better fit, we additionally used the “MuMIn” package (Bartoń 2024) to perform model selection and compared the results of the top‐ranked models with those of the simple model. Only seven of the 23 models in this study showed lower AIC and a significant improvement in model fit and were therefore used (Appendix S1). Some variables were transformed to log (*log*) or square root (*sqrt*) scales to obtain normally distributed residuals. Each linear mixed model was followed by post hoc analyses via the Tukey's honest significant difference test (Tukey test) to obtain the significant differences in the means of the colour metrics values between the time points within each population using the package “emmeans” (Lenth 2016) with the “regrid” function to back transform the transformed models. We generally reported the significant changes between the beginning of the experiment (minutes 0) and the end (minute 15). When no significant differences were found between these time points, we reported the earliest time point at which a significant change occurred. All pairwise contrasts are reported in Supporting Information.

## 4 Results

### 4.1 Colour Change on Achromatic Backgrounds

#### 4.1.1 Black Background

**FIGURE 4:**
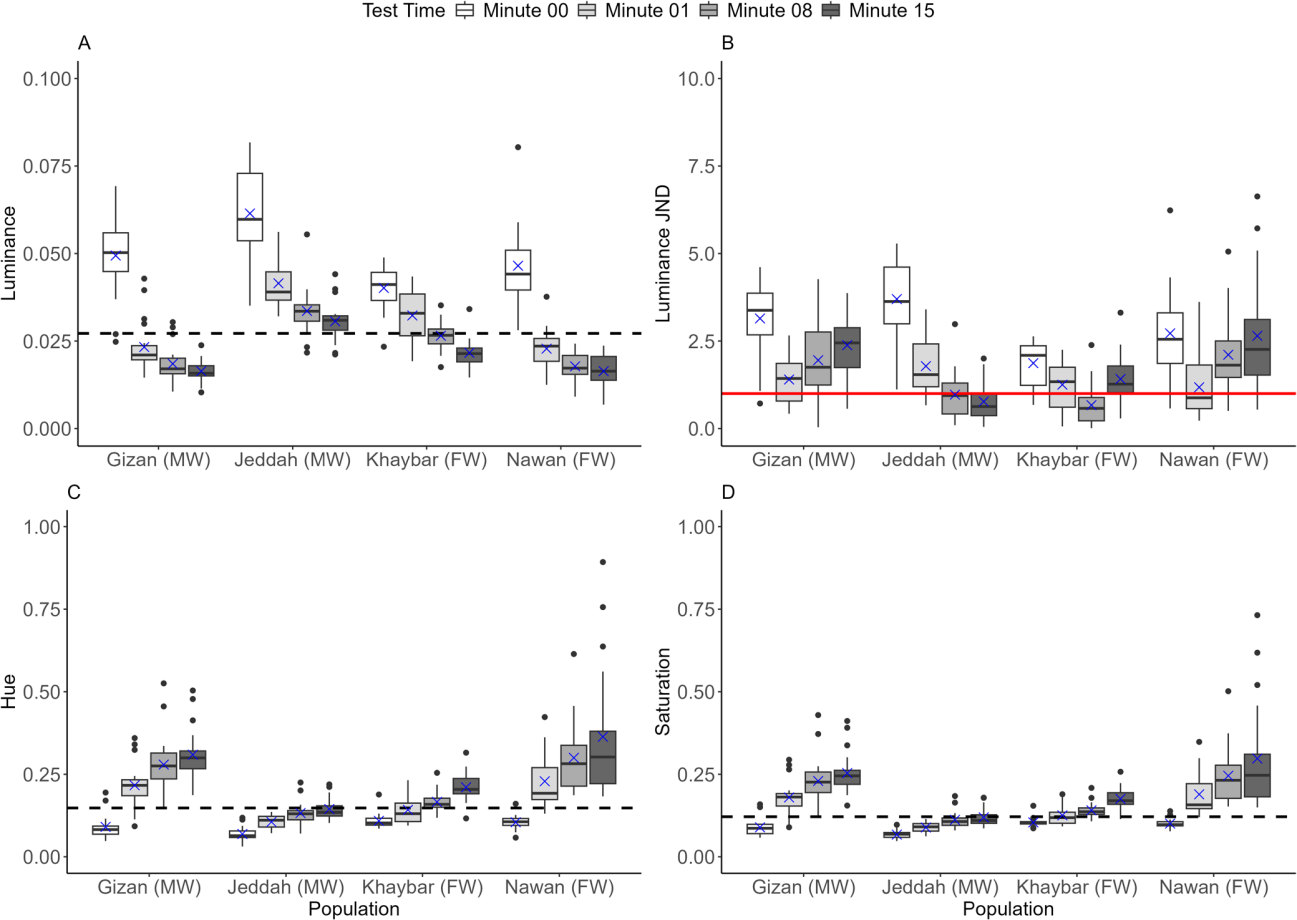
Changes in (A) the luminance, (B) luminance JND, (C) hue and (D) saturation within 15 min for all the populations (*n* = 20 per population) in the black background experiment. The boxes show the means (blue crosses), medians (black lines) and interquartile ranges. The whiskers represent the lowest and highest values within 1.5 × the interquartile ranges, and the dots represent the outliers. The horizontal black dashed lines represent the average values of the background luminance (A), hue (C) and saturation (D) for all the populations, and the horizontal solid red line represents the detection threshold of luminance contrast at 1 JND by the avian model (B). Following the population names, the letters in parenthesis FW and MW denote the freshwater and marine habitat types, respectively.

**FIGURE 5:**
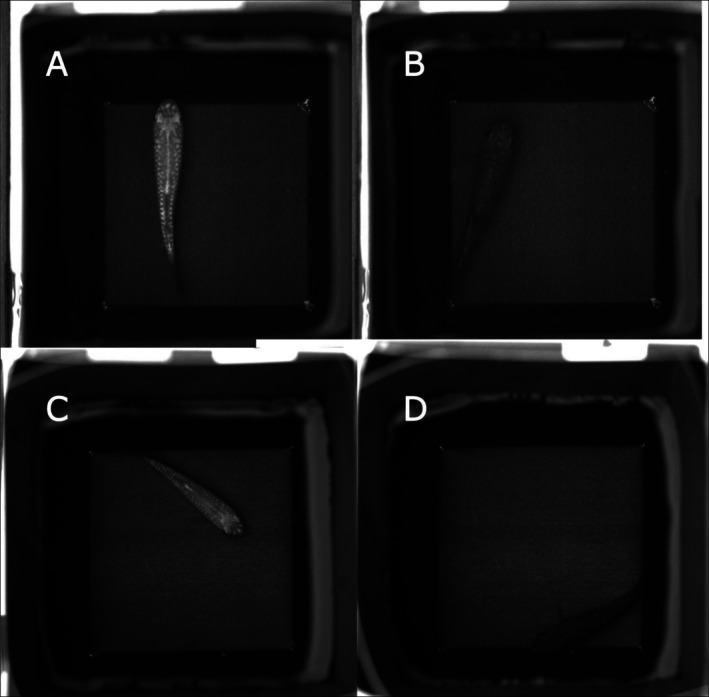
Unmodelled images (RGB images in grey mode) illustrating the changes in the brightness of an Arabian killifish from the marine population of Jeddah in the black experiment at minutes 0 (A) and 15 (B), as well as an individual from the freshwater population of Nawan at minutes 0 (C) and 15 (D).

There were significant differences between populations in luminance change indicated by the interaction between population and time (*log*(LMM): *F*(9,228) = 11.4243, *p* < 0.0001) (Figure 4A). The Tukey post hoc test between minutes 00–15 revealed highly significant changes for all the populations (*p* < 0.0001) (Figures 4A, 5). Luminance changes in the black background experiment occurred rapidly, within the first minute for all the populations (Tukey test: *p* < 0.0001), apart from the Khaybar population (Tukey test: *p* = 0.0003) (Figure S6), where luminance changed steadily. Changes between minutes 01–08 continued, with decreased significance observed in all the populations (i.e., not as highly significant as between minutes 00–01). However, no significant differences were observed between minutes 08–15, except for the Khaybar population, which retained a considerable change in luminance (Tukey test: *p* = 0.0019). There were also significant differences between populations in the changes of luminance JND revealed by the interaction between population and time (*sqrt*(LMM): *F*(9,228) = 11.721, *p* < 0.0001). Luminance JND decreased greatly within the first minute of the experiments for all the populations (Tukey test: *p* < 0.0001) except for the Khaybar population (Tukey test: *p* = 0.0283). However, apart from the population of Jeddah, which showed a significant and directional trend towards the detection threshold (i.e., JND = 1) (Tukey test: *p* < 0.0001) (Figure 4B), there was no significant improvement in camouflage for all the other populations by the end of the experiments (minutes 00–15).

There were significant differences between populations in hue change indicated by the interaction between population and time (*log*(LMM): *F*(9,228) = 11.1286, *p* < 0.0001) (Figure 4C). As in luminance changes, the Tukey post hoc test between minutes 00–15 revealed highly significant differences in hue for all the populations in the black background experiment (*p* < 0.0001), which changed faster within the first minute of the experiment for all the populations (*p* < 0.0001) except for the Khaybar population (*p* = 0.0023). The significant changes continued between minutes 01–08 but disappeared for all the populations between minutes 08–15, except for the Khaybar population, which retained the significant change (Tukey test: *p* = 0.0041). There were significant differences between populations in saturation change indicated by the interaction between population and time (*log*(LMM): *F*(9,228) = 12.4184, *p* < 0.0001) (Figure 4D). As in luminance and hue changes, the Tukey post hoc test between minutes 00–15 revealed highly significant differences in saturation for all the populations (*p* < 0.0001), which were faster within the first minute of the experiment for all the populations (*p* < 0.0001) except for the Khaybar population (*p* = 0.0181). There was no significant difference for all the populations between minutes 08–15, except for the Khaybar population, which retained the significant change (Tukey test: *p* = 0.0014). There were no significant effects of fish length and sex on all colour metrics in the black background experiment (Table S2).

#### 4.1.2 White Background

**FIGURE 6:**
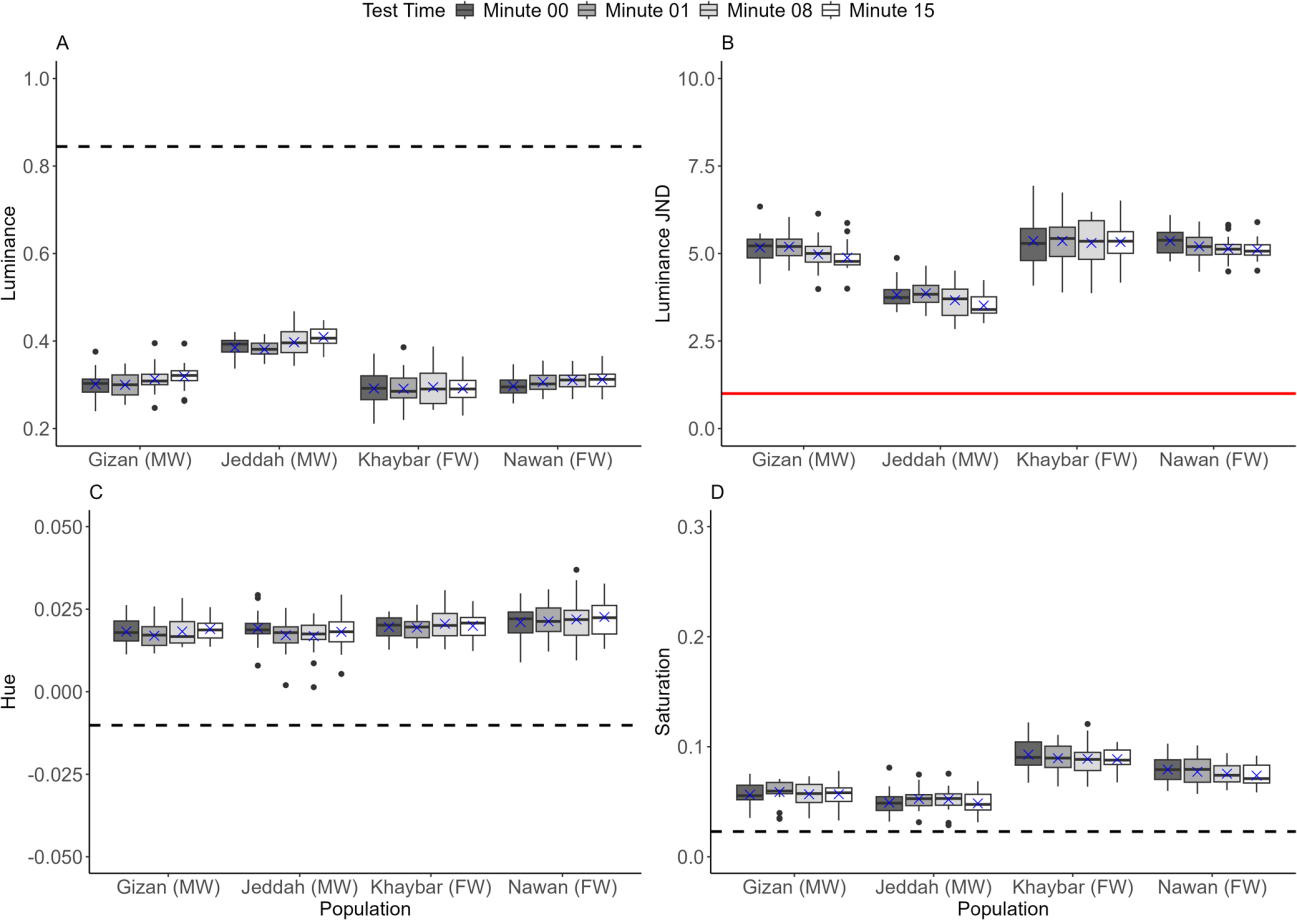
Changes in (A) the luminance, (B) luminance JND, (C) hue and (D) saturation within 15 min for all the populations (*n* = 20 per population) in the white background experiment. The boxes show the means (blue crosses), medians (black lines) and interquartile ranges. The whiskers represent the lowest and highest values within 1.5 × the interquartile ranges, and the dots represent the outliers. The horizontal black dashed lines represent the average values of the background luminance (A), hue (C) and saturation (D) for all the populations, and the horizontal solid red line represents the detection threshold of luminance contrast at 1 JND by the avian model (B). Following the population names, the letters in parenthesis FW and MW denote the freshwater and marine habitat types, respectively.

**FIGURE 7:**
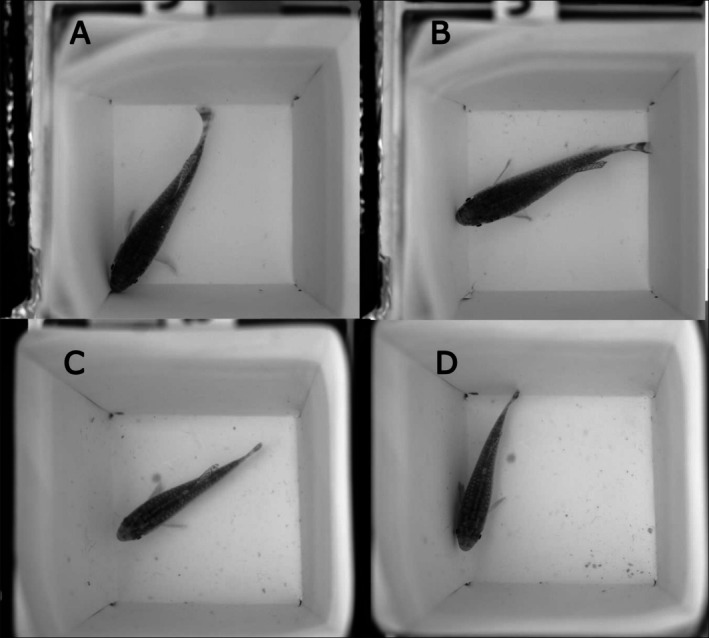
Unmodelled images (RGB images in grey mode) illustrating the changes in the brightness of an Arabian killifish from the marine population of Gizan in the white experiment at minutes 0 (A) and 15 (B), as well as an individual from the freshwater population of Khaybar at minutes 0 (C) and 15 (D).

There were significant differences between populations in luminance change to the white background revealed by the interaction between population and time (LMM: *F*(9,228) = 2.251, *p* = 0.0198) (Figure 6A). In contrast to the black background experiment, luminance changes in the white background experiment occurred mainly between minutes 00 and 15 for all the populations except the Khaybar population, in which luminance did not change significantly (Tukey test between minutes 00–15 in the populations of [Gizan: *p* = 0.0079], [Jeddah: *p* = 0.0001], [Nawan: *p* = 0.0343] and [Khaybar: *p* = 0.9998]; Figures 7, S7). Only the Jeddah population showed a significant change between minutes 01–08 (Tukey test: *p* = 0.0166) due to the decreased luminance within the first minute which increased afterwards (Figure 6A). There was a significant effect of fish length on luminance (LMM: *F*(1,74) = 15.018, *p* = 0.0002), but there was no significant effect of fish sex on luminance (Table S2). Additionally, the interaction between population and time revealed significant differences between populations in the changes of luminance JND (*log*(LMM): *F*(9,228) = 2.366, *p* = 0.0142) (Figure 6B). Luminance JND changes in the white background experiment mainly occurred by the end of the experiment for all the populations except for the Khaybar population (Tukey test between minutes 00–15 for the populations of Gizan: *p* = 0.0088; Jeddah: *p* < 0.0001; Nawan: *p* = 0.0466; Khaybar: *p* = 0.9998). There were significant effects of fish length (*log*(LMM): *F*(1,73) = 5.847, *p* = 0.018) and fish sex on luminance JND (*log*(LMM): *F*(1,73) = 6.038, *p* = 0.016) by which larger fish had higher JND, and males had higher JND. Additionally, there was a significant effect of the interaction between fish length and fish sex on luminance JND (*log*(LMM): *F*(1,73) = 5.290, *p* = 0.0243), where larger males had lower luminance JND and smaller females had lower luminance JND.

There were significant differences between populations in hue change indicated by the interaction between population and time (LMM: *F*(9,228) = 2.293, *p* = 0.0176). The Tukey post hoc test between time points indicated that the significance observed in hue changes in the white background experiment was due to changes in the Jeddah population between minutes 00–01 (*p* = 0.0117) and 00–08 (*p* = 0.003) and in the Gizan population between minutes 01–15 (*p* = 0.0155), but no significant differences were observed by the end of the experiments (Figure 6C). Additionally, there was no significant effect of fish length on hue in the white background experiment (Table S2), but there was a significant effect of fish sex on hue (LMM: *F*(1,73) = 6.579, *p* = 0.012) where males had higher hue than females. Additionally, there was a significant effect of the interaction between fish length and fish sex on hue (LMM: *F*(1,73) = 7.599, *p* = 0.007) where larger males had lower hue and smaller females had lower hue. There were significant differences between populations in saturation change, indicated by the interaction between population and time (*sqrt*(LMM): *F*(9,225) = 2.576, *p* = 0.007). The Tukey post hoc test between time points indicated that the statistical significance observed in saturation changes was due to changes in the Jeddah population between minutes 01–15 and 08–15 (*p* = 0.0067 and *p* = 0.0323, respectively) and in the Khaybar and Nawan populations between minutes 00–15 (*p* = 0.0067 and *p* = 0.0323, respectively). However, no significant differences were observed by the end of the experiments for the other populations (Figure 6D). There was a significant effect of fish length on saturation (*sqrt*(LMM): *F*(1,75) = 14.501, *p* = 0.0002). Additionally, there was an effect of the interaction between fish length and test time on saturation (*sqrt*(LMM): *F*(3,225) = 3.639, *p* = 0.0135) where the relationship between length and saturation changed over time, with larger individuals tending to show relatively lower saturation at minute 15 compared with earlier time points.

### 4.2 Colour Change on Chromatic Backgrounds

The findings consist of complex responses to three background colours, each of which has analyses of five colour metrics. The results of luminance, hue, saturation, colour JND and luminance JND are summarised in Table 1.

**TABLE 1:** Summary of the statistical analyses of the luminance, hue, saturation, colour JND and Luminance JND changes in the chromatic background experiments (beige, brown and green) for individuals from the four populations; freshwater (FW) and marine (MW).

Background colour	Population				Colour metric
	Gizan (MW)	Jeddah (MW)	Khaybar (FW)	Nawan (FW)	
Beige	✗	✔	✗	✗	Luminance
	✔ mala	✗	✗	✗	Hue
	✗	✔	✗	✔	Saturation
	✗	✔	✗	✔	Colour JND
	✗	✔	✗	✗	Luminance JND
Brown	✔	✗	✗	✗	Luminance
	✗	✔ mala	✗	✗	Hue
	○	✔	○	✗	Saturation
	✔	✗	○	✗	Colour JND
	✔	✗	✗	✗	Luminance JND
Green	✔	○	✗	✗	Luminance
	✔ mala	✔	✗	✗	Hue
	✔	✗	✗	○ M	Saturation
	✔	✔ mala	○	✗	Colour JND
	✔	○	✗	✗	Luminance JND

*Note:* Ticks (✔) indicate a significant difference (Tukey test: *p* < 0.05) in colour metric values between minutes 00 and 15, and crosses (✗) indicate that no significant changes were observed during the experiments. Circles (○) indicate that a significant difference was observed between two time points at the beginning or in the middle of the experiments. While (M) means that the statistical significance was marginal (Tukey test: *p*≈0.05). Some statistically significant changes in hue were not directed towards the hue values of the backgrounds (Figures 8B, 9B, 10B) and colour JND increased (Figure 10D), which might be likely maladaptive (mala). Each shade of the orange colour represents one of the results categories mentioned above.

### 4.3 Beige Background

**FIGURE 8:**
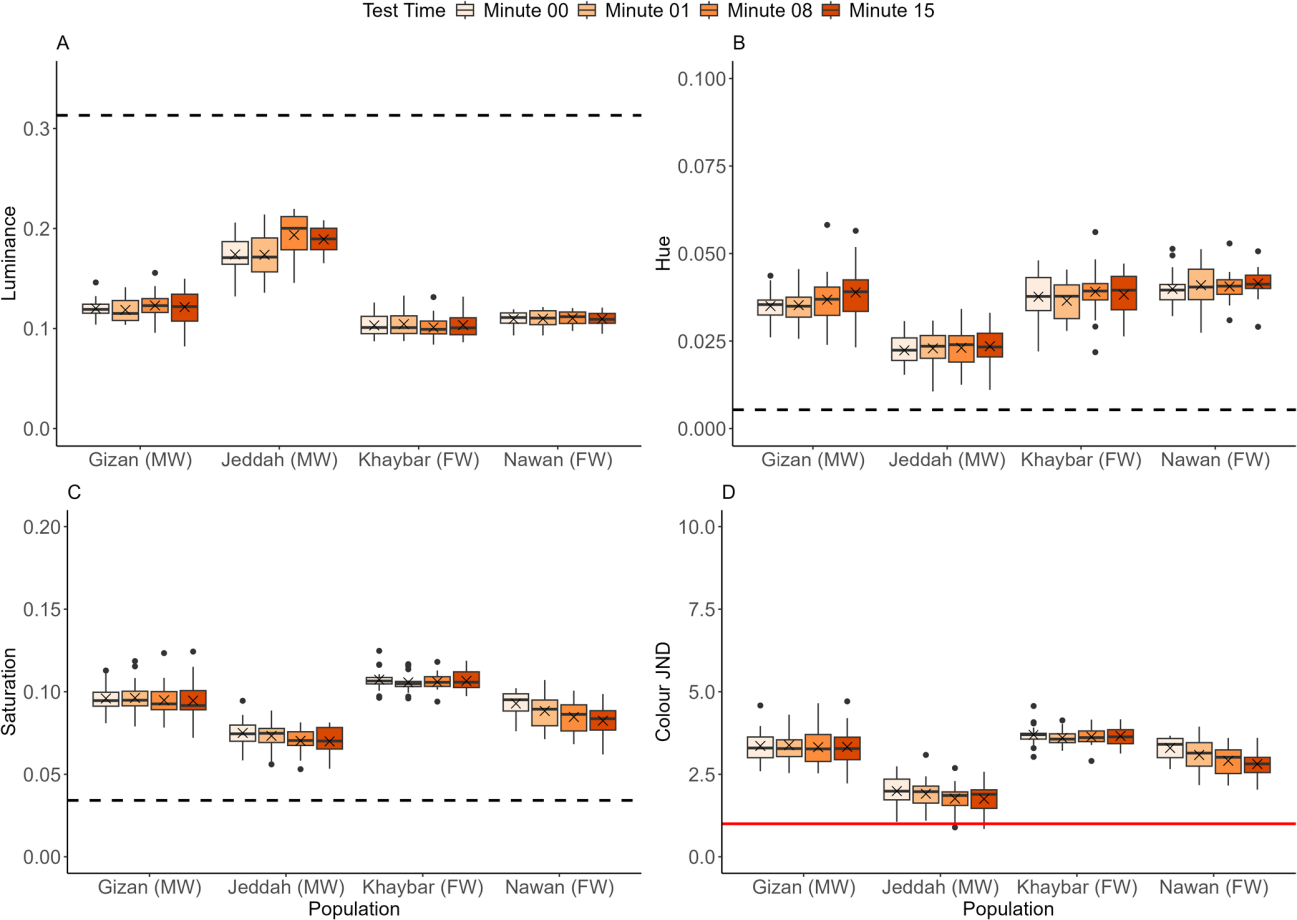
Changes in (A) the luminance, (B) hue, (C) saturation and (D) colour JND within 15 min for all the populations (*n* = 20 per population) in the beige background experiment. The boxes show the means (black crosses), medians (black lines) and interquartile ranges. The whiskers represent the lowest and highest values within 1.5 × the interquartile ranges, and the dots represent the outliers. The horizontal black dashed lines represent the average values of the background luminance (A), hue (B) and saturation (C) for all the populations, and the horizontal solid red line represents the detection threshold of colour contrast at 1 JND by the avian model (D). Following the population names, the letters in parenthesis FW and MW denote the freshwater and marine habitat types, respectively.

There were significant differences between populations in luminance change indicated by the interaction between population and time (*sqrt*(LMM): *F*(9,228) = 3.779, *p* = 0.0002). The pairwise Tukey post hoc test indicated that the Gizan, Khaybar and Nawan populations did not change luminance (Figure 8A). The Jeddah population showed statistically significant changes by the end of the experiment (*p =* 0.0005). There were significant differences between populations in the changes of luminance JND, demonstrated by the interaction between population and time (LMM: *F*(9,228) = 2.971, *p* = 0.0023). However, when further investigating this using the pairwise Tukey post hoc test between minutes, the Gizan, Khaybar and Nawan populations showed no significant changes in luminance JND, but the Jeddah population did (Tukey test between minutes 00–15: *p =* 0.0034) (Figure S8A). There was a significant effect of fish length on luminance (*sqrt*(LMM): *F*(1,74) = 9.772, *p* = 0.0025) and on luminance JND (LMM: *F*(1,74) = 8.914, *p* = 0.0038), but no significant effect was observed of fish sex on both metrics (Table S2).

There were no significant differences between populations in hue change as shown by the interaction between population and time (LMM: *F*(9,228) = 1.096, *p* = 0.3667) (Figure 8B). However, there was a significant effect of fish length on hue (LMM: *F*(1,74) = 8.100, *p* = 0.0057), and no significant effect of fish sex on hue (Table S2). There were significant differences between populations in saturation change indicated by the interaction between population and time (*log*(LMM): *F*(9,228) = 4.768, *p* < 0.0001) (Figure 8C). The pairwise Tukey post hoc test indicated that only the Jeddah and Nawan populations changed saturation significantly by the end of the experiments (*p* = 0.0001 and *p* < 0.0001, respectively). There was no significant effect of fish sex on saturation (Table S2), but there was a significant effect of the interaction between fish population and fish sex on saturation at baseline (*log*(LMM): *F*(3,72) = 8.350, *p* < 0.0001), which indicated that sex‐related differences in saturation were not consistent among populations.

There were significant differences between populations in the changes of colour JND shown by the interaction between population and time (*sqrt*(LMM): *F*(9,228) = 3.862, *p* = 0.0001). Only the Jeddah and Nawan populations showed highly significant changes in colour JND values (Tukey test: *p =* 0.0003 and *p* < 0.0001, respectively), whereas the Gizan and Khaybar populations showed no significant differences. Nevertheless, all individuals from all the populations in the beige background experiment were chromatically detectable, and only the Jeddah population closely matched the beige background (Figure 8D). Furthermore, there was a significant effect of fish sex on colour JND (*sqrt*(LMM): *F*(1,72) = 4.761, *p* = 0.0323), by which males showed higher colour JND. In addition, there was a significant effect of the interaction between fish population and fish sex on colour JND at baseline (*sqrt*(LMM): *F*(3,72) = 13.830, *p* < 0.0001), which indicated that sex‐related differences in colour JND were not consistent among populations.

### 4.4 Brown Background

**FIGURE 9:**
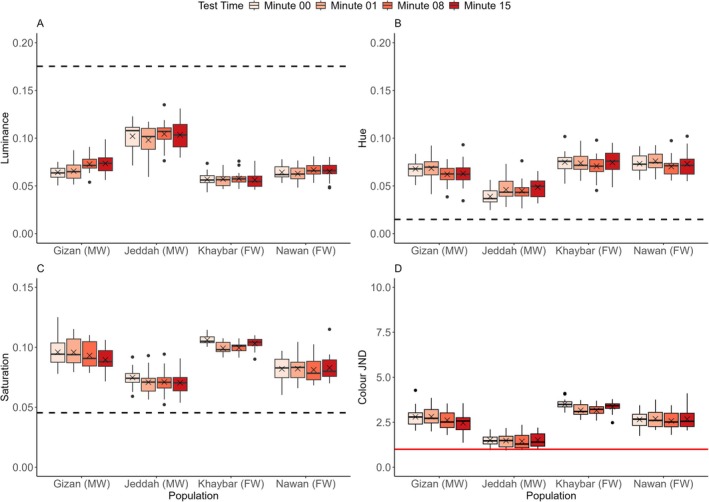
Changes in (A) the luminance, (B) hue, (C) saturation and (D) colour JND within 15 min for all the populations (*n* = 20 per population) in the brown background experiments. The boxes show the means (black crosses), medians (black lines) and interquartile ranges. The whiskers represent the lowest and highest values within 1.5 × the interquartile ranges, and the dots represent the outliers. The horizontal black dashed lines represent the average values of the background luminance (A), hue (B) and saturation (C) for all the populations, and the horizontal solid red line represents the detection threshold of colour contrast at 1 JND by the avian model (D). Following the population names, the letters in parenthesis FW and MW denote the freshwater and marine habitat types, respectively.

There was a non‐significant trend for differences between populations in luminance change indicated by the interaction between population and time (*sqrt*(LMM): *F*(9,228) = 1.870, *p* = 0.0574). The pairwise Tukey post hoc test indicated that the Jeddah, Khaybar and Nawan populations did not change luminance (Figure 9A). However, the marine population of Gizan showed significant changes in luminance by the end of the experiments (Tukey test: *p* = 0.0006). There were marginally significant differences between populations in the changes of luminance JND displayed by the interaction between population and time (LMM: *F*(9,228) = 1.936, *p* = 0.0480). The Tukey post hoc test between minutes showed that the Jeddah, Khaybar and Nawan populations exhibited no significant changes in luminance JND except for the Gizan population (Tukey test between minutes 00–15: *p* = 0.0004) (Figure S8B). Moreover, there were no significant effects of fish length and fish sex on luminance and luminance JND in the brown background experiment (Table S2).

There were significant differences between populations in hue change demonstrated by the interaction between population and time (*log*(LMM): *F*(9,228) = 4.715, *p* < 0.0001) where the Gizan, Khaybar and Nawan populations did not change hue (Figure 9B) compared with the Jeddah population, which showed significant changes by the end of the experiment (Tukey test: *p* < 0.0001). There was a significant effect of fish length on hue (*log*(LMM): *F*(1,74) = 6.696, *p* = 0.0116) and no significant effect of fish sex on hue (Table S2). There were significant differences between populations in saturation change indicated by the interaction between population and time (LMM: *F*(9,225) = 2.986, *p* = 0.0022) (Figure 9C). The pairwise Tukey post hoc test indicated that in the brown background experiment, only the marine Gizan and Jeddah populations changed saturation significantly (minutes 01–15: *p* = 0.0276 and minutes 00–15: *p* = 0.0039), and the freshwater population of Khaybar showed only a significant change at the beginning of the experiments (minutes 00–01: *p* < 0.0001). Furthermore, there was no significant effect of fish length on saturation in the brown background experiment (Table S2), but there were significant effects on saturation by the interaction between fish length and fish population at baseline (LMM: *F*(3,72) = 4.186, *p* = 0.008), and the interaction between fish length and test time (LMM: *F*(3,225) = 3.248, *p* = 0.0226). These results indicated that length effects on saturation were not consistent among populations at baseline, but generally the relationship between length and saturation changed over time, with larger individuals tending to show relatively lower saturation at minute 15 compared with earlier time points.

There were significant differences between populations in the changes of colour JND indicated by the interaction between population and time (*log*(LMM): *F*(9,228) = 2.381, *p* = 0.0136). However, only the Gizan population showed a significant shift in colour JND by the end of the experiments (Tukey test: *p* = 0.0029). The Khaybar population changed only at the beginning of the experiment (Tukey test: *p* = 0.0272) (Figure 9D). As in the beige background experiment, all individuals from all the populations in the brown background experiment were chromatically detectable, and only the Jeddah population had more similarities to the brown backgrounds (Figure 9D). Furthermore, there were no significant effects of fish length and fish sex on colour JND in the brown background experiment (Table S2).

### 4.5 Green Background

**FIGURE 10:**
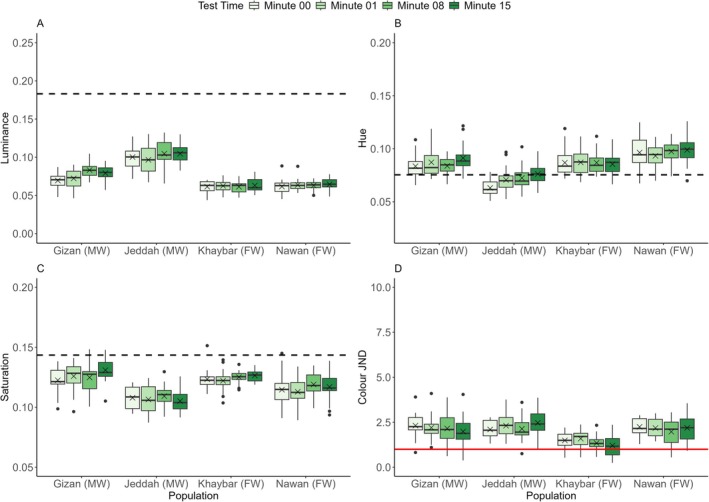
Changes in (A) the luminance, (B) hue, (C) saturation and (D) colour JND within 15 min for all the populations (*n* = 20 per population; only the Nawan population had 19 individuals at minute 08) in the green background experiments. The boxes show the means (black crosses), medians (black lines) and interquartile ranges. The whiskers represent the lowest and highest values within 1.5 × the interquartile ranges, and the dots represent the outliers. The horizontal black dashed lines represent the average values of the background luminance (A), hue (B) and saturation (C) for all the populations, and the horizontal solid red line represents the detection threshold of colour contrast at 1 JND by the avian model (D). Following the population names, the letters in parenthesis FW and MW denote the freshwater and marine habitat types, respectively.

There were significant differences between populations in luminance change revealed by the interaction between population and time (LMM: *F*(9,226.552) = 3.209, *p* = 0.0011). The pairwise Tukey post hoc test indicated that the Khaybar and Nawan populations did not change luminance (Figure 10A). However, the marine populations of Gizan and Jeddah showed significant changes in luminance, with the Gizan population showing changes by the end of the experiment (Tukey test: *p* = 0.0006). The Jeddah population showed significant changes in the middle of the experiment (Tukey test between minutes 01–08: *p* = 0.0057) (Figure 10A). There was a significant effect of fish length on luminance (LMM: *F*(1,73.351) = 4.172, *p* = 0.0447) and no significant effect of fish sex on luminance (Table S2). There were significant differences between populations in the changes of luminance JND indicated by the interaction between population and time (*sqrt*(LMM): *F*(9,226.207) = 3.063, *p* = 0.0018). When further investigating this using the pairwise Tukey post hoc test between minutes, the Khaybar and Nawan populations showed no significant changes in luminance JND (Figure S8C). However, the marine population of Gizan showed significant changes in luminance JND by the end of the experiments (Tukey test: *p* = 0.0008), whereas the Jeddah population displayed significant changes in the middle of the experiment (Tukey test between minutes 01–08: *p* = 0.0238). There was a significant effect of fish length on luminance JND (*sqrt*(LMM): *F*(1,72.998) = 4.861, *p* = 0.0306) and no significant effect of fish sex on luminance JND (Table S2).

There were significant differences between populations in hue change demonstrated by the interaction between population and time (*log*(LMM): *F*(9,226.588) = 3.243, *p* = 0.0010) (Figure 10B). The pairwise Tukey post hoc test indicated that the Khaybar and Nawan populations did not change the hue. However, the marine populations of Gizan and Jeddah showed significant changes by the end of the experiments (*p* = 0.0337 and *p* < 0.0001, respectively). There were no significant effects of fish length and fish sex on hue (Table S2). There were significant differences between populations in saturation change, indicated by the interaction between population and time (LMM: *F*(9,226.657) = 2.731, *p* = 0.0048) (Figure 10C). The pairwise Tukey post hoc test indicated that only the marine Gizan population changed saturation significantly by the end of the experiments (*p* = 0.0001), and only the freshwater Nawan population showed a marginally significant change in the middle of the experiments (minutes 01–08: *p* = 0.0462). Furthermore, there was no significant effect of fish length on saturation in the green background experiment (Table S2), but there was a significant effect on saturation by the interaction between fish length and fish population at baseline (LMM: *F*(3, 71.512) = 2.794, *p* = 0.046), which indicated that length effects on saturation were not consistent among populations at baseline.

There were significant differences between populations in the changes of colour JND exhibited by the interaction between population and time (LMM: *F*(9,227.034) = 3.314, *p* = 0.0008). The pairwise Tukey post hoc test between minutes revealed that the marine populations of Gizan and Jeddah changed colour JND significantly by the end of the experiments (*p* = 0.0267 and *p* = 0.0095, respectively), but the freshwater Khaybar population showed a change only between minutes 01–15 (*p =* 0.0060), and no differences were observed in the Nawan population. The avian model showed that some individuals from all the populations were undetectable on the green background (Figure 10D), possibly due to their natural green hues. Furthermore, there were no significant effects of fish length and fish sex on colour JND in the green background experiment (Table S2).

## 5 Discussion

In this study, we investigated the geographical variation in the capacity for achromatic and chromatic colour change among marine and freshwater *A. dispar* populations. This is the first study on colour change in a member of this ecologically interesting family, Aphaniidae, which serves as a baseline for future work on *A. dispar* and other Aphaniidae members. The findings indicated that the freshwater and marine populations of the Arabian killifish can change colour rapidly within a few minutes and showed increased ability for achromatic colour change (luminance) in response to the black and white substrates, compared with chromatic colour change in response to the beige, brown and green substrates. The marine populations appeared more capable of changing colour on chromatic backgrounds than the freshwater populations. Additionally, there was an interpopulation variation in colour change capabilities. Although all the populations exhibited the same directional responses to black backgrounds (i.e., became darker), the relative initial (after minute 1) and final (after minute 15) change rates varied among them (Figures S6, S7).

There are some possible explanations for the apparent differences in luminance change ability on the black backgrounds. First, fish differed in their luminance at the start of the experiment, and hence the quantity of pigments may have constrained the dispersion ability (i.e., the more pigments the fish had, the greater they displayed the darkening response). To some extent, this might be valid; the Jeddah population had the greatest starting luminance and a 30.69% relative shift to dark during the first minute. Furthermore, the populations of Gizan and Nawan had nearly similar average luminance at the beginning and also had similar relative change rates, and after one minute, both showed a change of more than 48%. The Khaybar population had the lowest luminance at the start of the experiment, perhaps due to a high quantity of melanosomes, and exhibited slower and weaker changes, decreasing luminance by 18.5% during the first minute and 44.9% by the end of the experiment (Figures S6, S7). Second, the variation in response to changing luminance may also be due to geographical variation in selective pressures, such as habitat brightness. In the white background experiment, all the populations showed a similar final increase in luminance (i.e., luminance at minute 15 relative to minute 00) ranging from 5.3%–6.6%, except the Khaybar population, which showed only a 0.95% increase (Figure S7). A combination of the two explanations appears reasonable; it could be that the populations of Gizan, Jeddah and Nawan may have evolved greater changing abilities but differed in rates due to morphological and/or physiological constraints. In contrast, the population of Khaybar might have diverged into a decreased capacity to alter luminance to black and white backgrounds. The Khaybar population inhabits dark brown basaltic springs, darkened by irrigation and vegetation, which may likely have reduced their changing response compared with the other populations. These findings raise the question of why the changes in the two directions (i.e., black vs. white) differ substantially in all the populations.

Although extremely black and white surfaces are uncommon in nature, the utilisation of extreme background brightness (i.e., very dark and very light) may disclose information about the limits of colour plasticity (Choi and Jang 2014). We observed significant within‐population differences in response to black and white backgrounds. All the populations changed luminance stronger and faster in the black background experiment than in the white one, with most changes occurring within the first minute in the black substrates and taking 8 to 15 min on the white backgrounds. The change in brightness to white has also proven challenging in other freshwater fish species, compared with brown and green backgrounds (Cox et al. 2009).

There are two non‐exclusive possibilities for the different responses to black and white backgrounds. First, aggregating pigments in fish can be challenging or constrained by their pigment quantity, and the high concentration of pigments within chromatophores can make the animals appear dark even when pigments are aggregated. Therefore, significant changes to white may only be possible through the removal of pigments (Sugimoto et al. 2005) during the process of shifting to morphological colour change (Alfakih et al. 2022). Second, white backgrounds are rare in nature, so the ability to match them may not have been favoured by selection (Choi and Jang 2014; Stevens, Lown, and Denton 2014; Smithers et al. 2018), or they may induce a stress response in the fish. We found that the Jeddah and Gizan populations slightly darkened their bodies upon being placed on the white backgrounds during the first minute. This darkening response was also found in other colour‐changing fish species (Da Silva et al. 2020). However, while pigment aggregation can be used to some extent for background matching, the killifish may likely employ it as a response to more predictable changes, such as seasonal changes (Caro et al. 2016).

Contrary to the white backgrounds, substrates with low brightness might be more common, and being on a dark background and shifting to a dark colouration may impair predator vision, resulting in reduced predation potential (Sumner 1934, 1935a, 1935b). Several lines of evidence demonstrated that different fish species prefer dark substrates more frequently (Brown and Thompson 1937; Bradner and McRobert 2001; Kjernsmo and Merilaita 2012; Smithers et al. 2018).

Despite being acclimatised to the same intermediate grey backgrounds, fish from the same population exhibited differences in luminance, hue and saturation values at the beginning (time‐point zero) of the black and white background experiments (i.e., there appears to be some instantaneous colour change). This difference was also consistently observed across all chromatic treatments. This is unlikely to be due to changes in pigmentation, but instead is probably caused by the reflective properties of the light‐reflecting cells in the fish's scales, especially leucophores (Goda and Kuriyama 2021), which may have mirrored some of the background colour. Consequently, fish on lighter backgrounds appeared lighter, while those on darker backgrounds appeared darker. This effect might serve as a passive mechanism for improving camouflage through matching the background (Hanlon and Messenger 2018), which the fish could naturally employ in the wild as an initial response to habitat heterogeneity over changing colour. As the fish were kept on the same background after this point, any subsequent colour change has to be due to changes in pigmentation.

The marine populations of Gizan and Jeddah appeared to be more capable of changing colour on chromatic backgrounds than the freshwater populations of Khaybar and Nawan (Table 1). We found no evidence for consistent differences between and within populations in chromatic changes (hue, saturation or even luminance), with some populations displaying changes in one colour metric and not in the other metrics. These results confirmed the previous observations made on four different populations of dwarf chameleons *B. transvaalense* where responses were not consistent in luminance and colour contrast against two different substrates (Stuart‐Fox et al. 2006). There are possibly two explanations for the differences in achromatic and chromatic colour change ability among and within populations. First, antipredator responses may vary due to differences in selective regime based on predator exposure, leading to population‐specific antipredator strategies (Magurran et al. 1993). The marine populations may have evolved a higher capacity for chromatic change due to higher diversity of predators in the Red Sea shores (Lieske and Myers 2004; Boland and Alsuhaibany 2020) compared with the freshwater populations, which may face fewer predators. To illustrate, a recent study suggests that relaxed predation pressure influenced the extent of colour change in the invasive Jackson's chameleon ( *Trioceros jacksonii xantholophus* ) in Hawaii (Whiting et al. 2022). This introduced chameleon changed colour during social interactions, and when presented with predator models, they changed colour in a way that rendered them more conspicuous—more contrasting against the background—in the non‐native Hawaiian habitats than the original African populations in their native environments (Whiting et al. 2022).

Second, colour change ability may be developmentally plastic or learned, with the differences emerging from prolonged exposure to multiple avian and aquatic predators in the marine environments, as compared with the freshwater populations. Females of *Aphaniops* members reach a standard length of approximately 2.5 cm within 3–4 months, while males are generally smaller at this age (Coad 2010; Freyhof et al. 2020). The mean standard length of the collected individuals in this study was 3.5 cm ± 0.67 (standard deviation), meaning that the fish collected were mature and likely had been exposed to their local predators for several months. The exposure to predators might have increased their chromatic response through learning from continuous prey–predator interactions (Kelley and Magurran 2003). Despite the fact that it is rarely discussed, at least one study on the zebrafish ( *Danio rerio* ) demonstrated some evidence of the role of learning in modulating colour change (Hatamoto and Shingyoji 2008). The first explanation that pertains to the marine populations evolving a higher capacity for chromatic changes would require strong and convergent selection to maintain the differences, given that molecular data suggested very little genetic divergence and probably continuity of gene flow between the marine and freshwater populations (Schunter et al. 2021), as in Nawan and Jeddah (Unpublished).

In both the achromatic and chromatic experiments, all adjustments in luminance and saturation were directed towards matching the corresponding background values. Some of the changes in hue for the marine populations of Gizan and Jeddah, however, deviated from the average hue values of the backgrounds (Table 1; Figures 8B, 9B, 10B). We also noted some significant fluctuations in luminance and saturation on the brown and green substrates that occurred at the beginning or middle of the experiments and then reversed (Table 1; Figures 9C, 10A,C). These oscillatory‐style changes are consistent with previous findings on a variety of colour‐changing species, including fish (Clarke and Schluter 2011; Whiteley et al. 2011; John et al. 2023) and amphibians (Kang et al. 2016), but their occurrence remains unresolved, though energetic expenditure on pigment maintenance during changing colour may play a partial role (Alfakih et al. 2022).

Our findings indicated that significant changes in luminance on the white and black backgrounds increased the fish's visual similarities to their previously mismatched substrates. However, while the improvement in camouflage in the white experiments was statistically significant, all individuals were highly detectable (Figure 6B). By the end of the experiments, however, all the populations in the black background experiment reached, on average, better matches to the black backgrounds, and some individuals were undetectable (< 1 JND). With the exception of the Jeddah population, which had the best match at the end of the experiments, all the other populations had the greatest concealment around the first or eighth minute (Figure 4B), despite all changing considerably in luminance. This occurred due to the fish “overshooting” and apparently becoming darker than their backgrounds (Figure 4A), leading the RNL model to determine them as less camouflaged than previous time points (Figure 4B). Luminance contrast is context‐dependent (van den Berg et al. 2020); therefore, it cannot be corroborated whether fish darker than the black substrates were detectable or well camouflaged because this would necessitate behavioural validation (van den Berg et al. 2020). Furthermore, the visual capability of the killifish is not known as they might perceive luminance and colour differently than the avian visual model used. Generally, darkening body colour and being on dark substrates may reduce predation likelihood (Sumner 1934, 1935a, 1935b).

The potential significance of hue and saturation changes in modifying achromatic camouflage cannot be ruled out, but changes in these colour attributes may be byproducts of changes in luminance (Stevens, Lown, and Wood 2014; John et al. 2023) and might even be perceptually small, contributing little to overall achromatic concealment (Stevens, Lown, and Wood 2014). It has been found that transitioning from dark to white colour was accompanied by an increase in saturation (Kang et al. 2016). However, how different types of chromatophores operate concurrently is not well known (Bagnara and Hadley 1973), and histological studies are required to describe chromatophore configuration during colour change.

In all chromatic experiments, there were statistically significant changes in chromatic contrasts (i.e., colour JNDs) for some populations. By closely looking at the data, those modifications were mainly accompanied by significant changes in saturation. In other animals, such as the Japanese tree frog ( *Hyla japonica* ), saturation was shown to be more effective than hue for achieving background matching (Choi and Jang 2014; Kang et al. 2016). Interestingly, our findings suggest that hue changes did not fully contribute to improved chromatic camouflage. For example, in the green experiment, the Jeddah population exhibited significant directional hue shifts (Figure 10B), but these shifts were accompanied by a negative effect and reduced camouflage (Figure 10D). However, determining why changes in hue and saturation remain operational is challenging, given their negligible significant contribution to concealment. The activities in chromatophores underlying these changes may have persisted as evolutionary vestiges when selection pressures were alleviated or absent (Rayner et al. 2022), or equally valid, they are novel responses under positive selection. Alternatively, altering colour simultaneously to match multiple colour attributes in the visual environment (e.g., brightness, hue and saturation) may be physiologically constrained (Choi and Jang 2014). Although changes in chromatic experiments were complicated, and some may appear to be maladaptive, they may be adaptive in another context (Waring 1963; Norris and Lowe 1964), for example, inducing shifts to morphological colour change. Nonetheless, all individuals had high luminance JNDs which were achromatically visible in the chromatic experiments (Figure S8A–C), despite some achromatic modifications exhibited by the marine populations. The ineffective colour change could have been caused by the fish gathering more visual information from the walls of the photography containers rather than the basal substrates, which might have been perceived as darker. As a result, their colour responsiveness might have been altered, which may also explain why the fish became darker than the black backgrounds in the achromatic experiments. However, the sensory limitations and the use of photoreceptors to estimate colour and luminance differences are not known in the Arabian killifish. Studies on these aspects are lacking in many animals, which need more investigation using behavioural and neurophysiological approaches (Bullough et al. 2023).

While changes in hue and saturation of chromatophores may impact the receiver's perception of colours, their physical mechanisms are more challenging to comprehend than changes in luminance, which are primarily attributable to melanosome dispersion and aggregation. The findings suggest that the luminance response to backgrounds is the primary change within visual environments mediated by physiological colour change, whereas chromatic responses involving independent hue and saturation changes may require an extended period and adjustments mediated by morphological colour change. In many animals, including fish species, brightness changes were reported to be easier than the actual colour changes (e.g., Whiteley et al. 2011; Da Silva et al. 2020), which appeared to be a primary response for camouflage that may impede predator detection (Kang et al. 2016). Indeed, many animals rely on achromatic information to recognise small and moving elements (Osorio et al. 1999), and hue change may be less critical than brightness change for successful camouflage (Stuart‐Fox et al. 2006). The Arabian killifish is highly mobile and small, so rapid achromatic responses may be more advantageous for impeding visual detection.

Fish length affected some initial colour aspects in certain treatments, including luminance and luminance JND in the white, beige and green experiments, hue in the beige and the brown experiments, and saturation in the white experiments. Generally, at the beginning of the experiments, larger individuals appeared darker, with higher hue and saturation values than the smaller ones, and hence were more detectable. Colour change strengthens the defensive abilities of small fish species that are vulnerable to predators (Cox et al. 2009), and juvenile fish may also benefit more from this plasticity (Sköld et al. 2016). The Arabian killifish occupies shallow water, and so we expect colour plasticity to persist throughout all life stages, unlike species, for example, that use colour change for mimicry, where only small individuals display colour change (Cheney et al. 2008). Changing colour is advantageous for increasing fitness, especially in ephemeral freshwater streams, so that they can reach maturity and mate before the drought season.

Fish sex affected some initial colour aspects in certain treatments, where males had higher luminance JND and hue in the white experiment and higher colour JND in the beige experiment. For animals that rely on colour adjustments for concealment, selection should reduce within‐population variation in colour change unless trade‐offs from other alternative behavioural strategies and differential physiological costs (Alfakih et al. 2022) or stress are involved (Gibson et al. 2009). Although we did not analyse the effects of sex on colour change as it is out of scope, we expect a lack of sex‐based variation in colour change in our context as the killifish live in mixed‐sex shoals, but there may be context‐dependent and transient differences in colour change between the sexes (e.g., during nuptial displays).

Nevertheless, when Nardo in 1827 described the genus *Aphanius* (including *Aphaniops*), there was no mention of the meaning of this generic name. The name *Aphanius* comes from Greek, which means inconspicuous (Wildekamp 1993; Esmaeili et al. 2020). In this research, we provided evidence that the Arabian killifish can be visually inconspicuous by means of background matching via rapid colour change. This inconspicuousness may be widespread among Aphaniidae, which might have led Nardo to choose the genus name, *Aphanius*. The Aphaniidae family is well known for adapting to various environmental factors, especially salinity and temperature, which appear to be conserved traits among many species in the family (Freyhof and Kottelat 2007; Freyhof et al. 2020). Whether colour change persists among the Aphaniidae members is yet to be determined. To the best of our knowledge, there are only two studies that mentioned the variation in colouration (i.e., brightness, not the patterns on the flanks) among populations of two species, the Mediterranean killifish ( *Aphanius fasciatus* ) (Cavraro et al. 2021) and the Arabian Gulf killifish (*Aphaniops stoliczkanus*) (Bidaye et al. 2023) by which the variation has been loosely interpreted as an interspecific variation due to habitat differences and not a plastic response to the different habitats (i.e., via colour change). The results presented in this research are not in agreement with that interpretation because unpublished molecular data suggest that the Gizan population may represent a new cryptic species *A. cf. dispar* when compared with populations from Jeddah, Khaybar and Nawan, which appear to belong to a single species, *A. dispar* . Notably, both genetic units exhibit the ability to change colour. It is possible, therefore, that *A. fasciatus* and *A. stoliczkanus* also possess this ability, which may be a conserved trait among other members of the Aphaniidae family.

Given the aforementioned, it would therefore be intriguing to study the evolution of colour change among and within members of the Aphaniidae family. To date, comparative phylogenetic studies to investigate the evolution of colour change seem limited only to Chamaeleonidae (Stuart‐Fox and Moussalli 2008; Dollion 2019). Several molecular studies on the Aphaniidae have covered most members, especially those that utilised mitochondrial DNA (e.g., Esmaeili et al. 2020). Therefore, a comparative phylogenetic analysis utilising a time‐calibrated phylogeny approach would strengthen the study of the evolution of colour change in Aphaniidae.

## 6 Conclusion

In this study, we provided the first evidence of the geographical variation in colour plasticity among populations of a non‐migratory euryhaline species of fish, *A dispar*, residing in two extreme ecosystems that differ in the visual backgrounds and potentially in predator diversity. Despite genetic similarity between the investigated populations (Unpublished), especially between the marine Jeddah and freshwater Nawan, a substantial variation in colour plasticity, particularly in chromatic responses, was observed. Counter to our prediction, the freshwater population of Nawan, which inhabited more varied visual environments, showed less ability to change colour compared with the marine populations in Gizan and Jeddah, which inhabited more homogeneous environments. The variation in responses observed in our study may reflect the influence of factors other than habitat visual heterogeneity, including the potential differences in predator communities. However, the effects of predator abundance and diversity on colour change were not directly assessed in this study and should be studied to determine whether predation pressure differs between marine and freshwater environments. Visual characteristics may nevertheless also be crucial in shaping the capacity for colour change. Saudi Arabia's freshwater streams are generally more visually diverse than coastal areas, exhibiting numerous achromatic and chromatic patches at fine‐scale levels. However, the habitats of the northernmost freshwater population of Khaybar where springs among basaltic volcanic rocks are uniformly dark brown due to irrigation and arboreal activities may have contributed to the lower white response compared with the southern freshwater counterpart. Due to a shortage of ecological data, it is challenging to disentangle the interaction effects of predator diversity and habitat heterogeneity on interpopulation variation in physiological colour change. We recommend that future research on physiological colour change consider (1) longitudinal studies on colour change in comparison with temporal habitat changes, which are almost non‐existent in the literature; (2) removing the differences in colouration between populations by enabling animals to undergo morphological colour change for extended periods in uniform settings (e.g., intermediate visual habitat); or (3) using laboratory‐bred individuals under uniform conditions.

## Author Contributions

**Ateah Alfakih:** conceptualization (lead), data curation (lead), formal analysis (lead), funding acquisition (lead), methodology (lead), visualization (lead), writing – original draft (lead), writing – review and editing (equal). **Nicola J. Nadeau:** formal analysis (supporting), supervision (lead), writing – review and editing (equal). **Penelope J. Watt:** formal analysis (supporting), supervision (lead), writing – review and editing (equal).

## Funding

This work was supported by Albaha University, which granted a PhD scholarship to AA (2019/40245343).

## Conflicts of Interest

The authors declare no conflicts of interest.

## Supporting information


**Appendix S1:** ece373005‐sup‐0001‐AppendixS1.docx.

## Data Availability

All data and codes, full pairwise contrast results as well as the Supporting Information for visual modelling were deposited in FigShare: https://doi.org/10.6084/m9.figshare.29211149.
