# Supplementary material for: The Geographical Variation of Colour Change in the Arabian Killifish (Aphaniops dispar Sensu Lato) From Freshwater and Marine Ecosystems
Source: Ecol Evol. 2026 Feb 17;16(2):e73005. doi: 10.1002/ece3.73005 (PMC12912887; doi:10.1002/ece3.73005)
Supplement: Supplementary file 1 — Appendix S1: ece373005‐sup‐0001‐AppendixS1.docx. [file ECE3-16-e73005-s001.docx]

**Introduction**


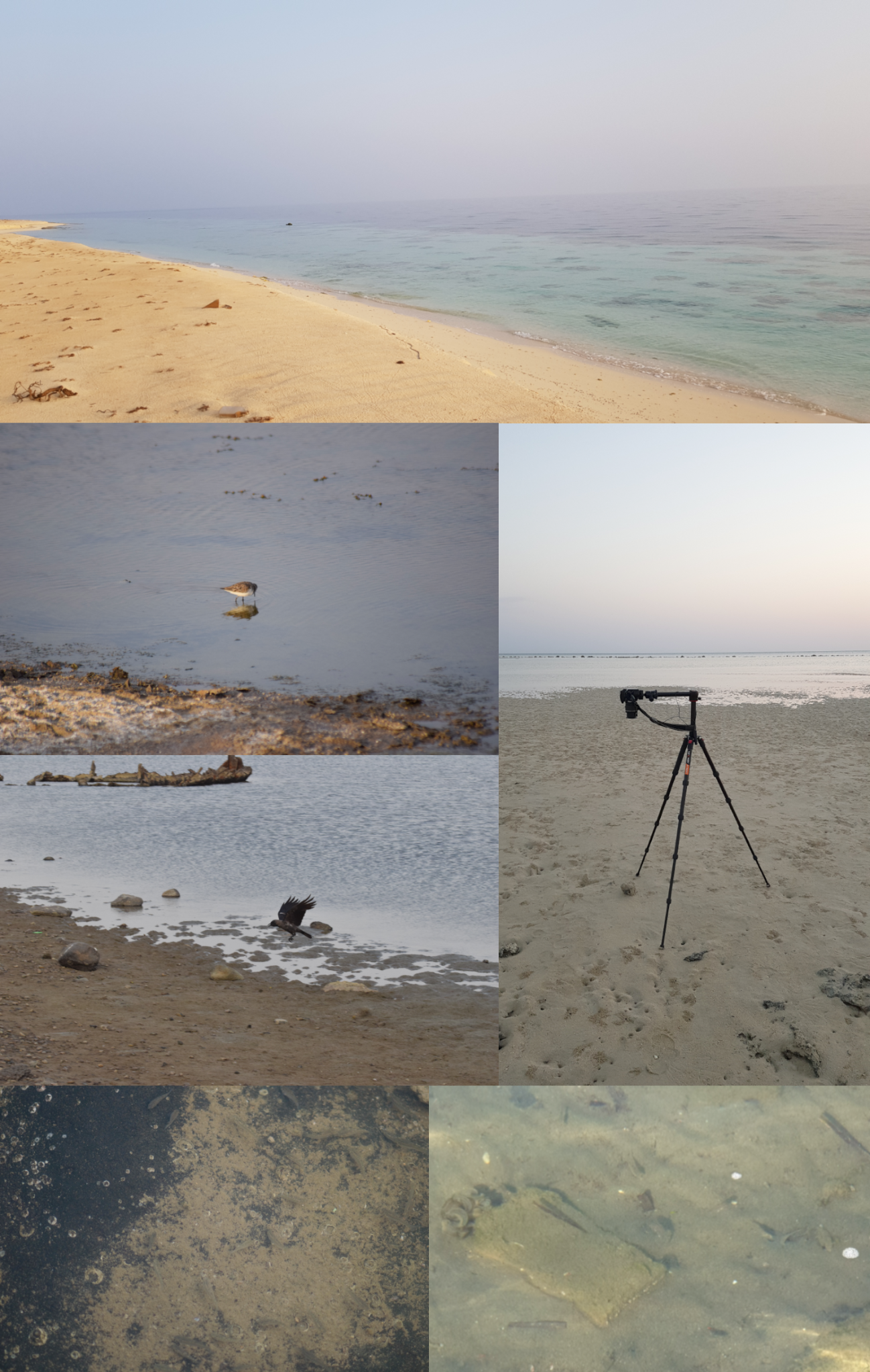


**Figure S1:** A combination of images shows the visual substrates at different marine locations where the Arabian killifish were observed along the Saudi Arabian Red Sea.


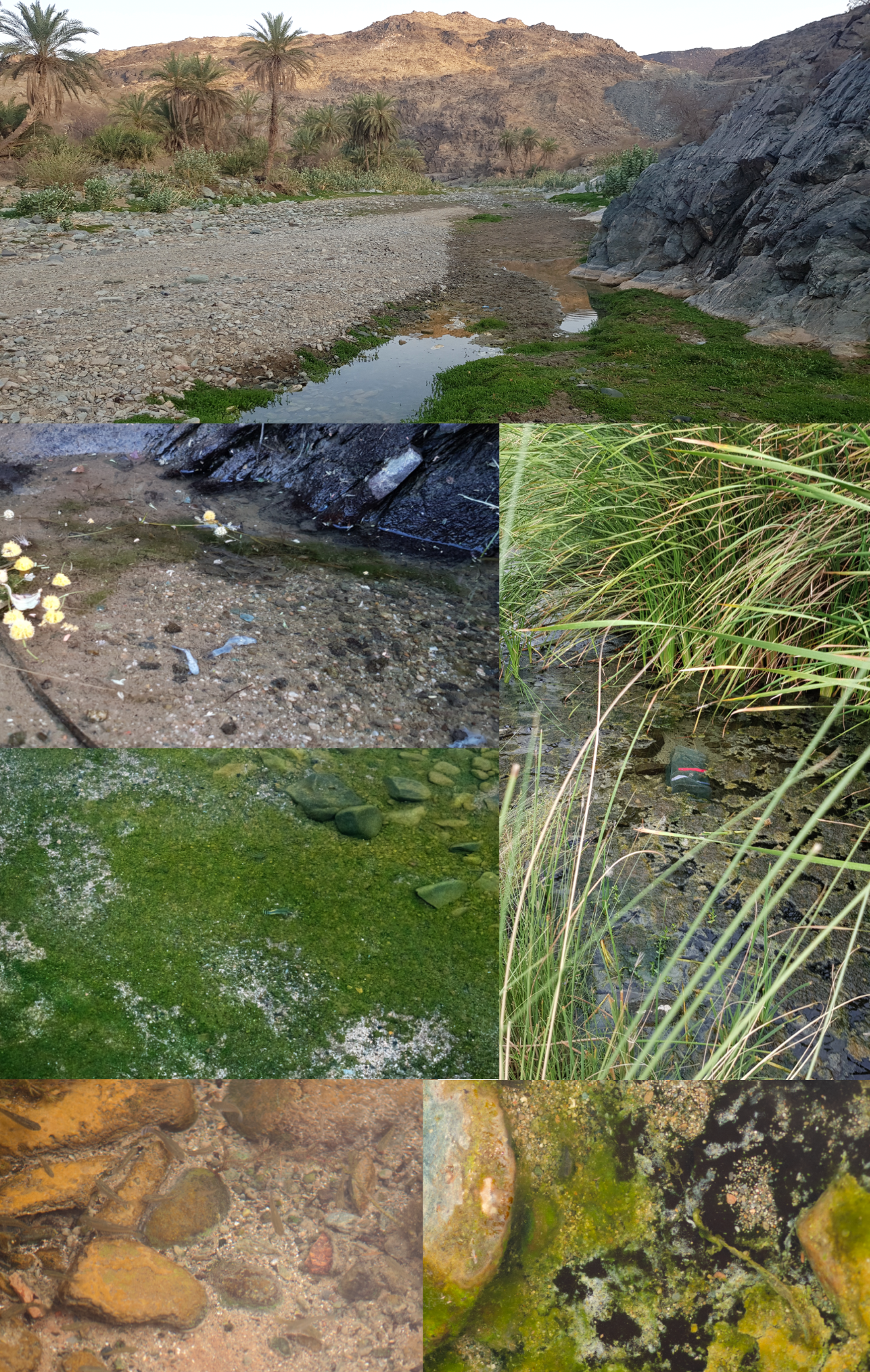


**Figure S2:** A combination of images shows the visual substrates at different freshwater locations where the Arabian killifish were observed in the western wadis of Saudi Arabia.


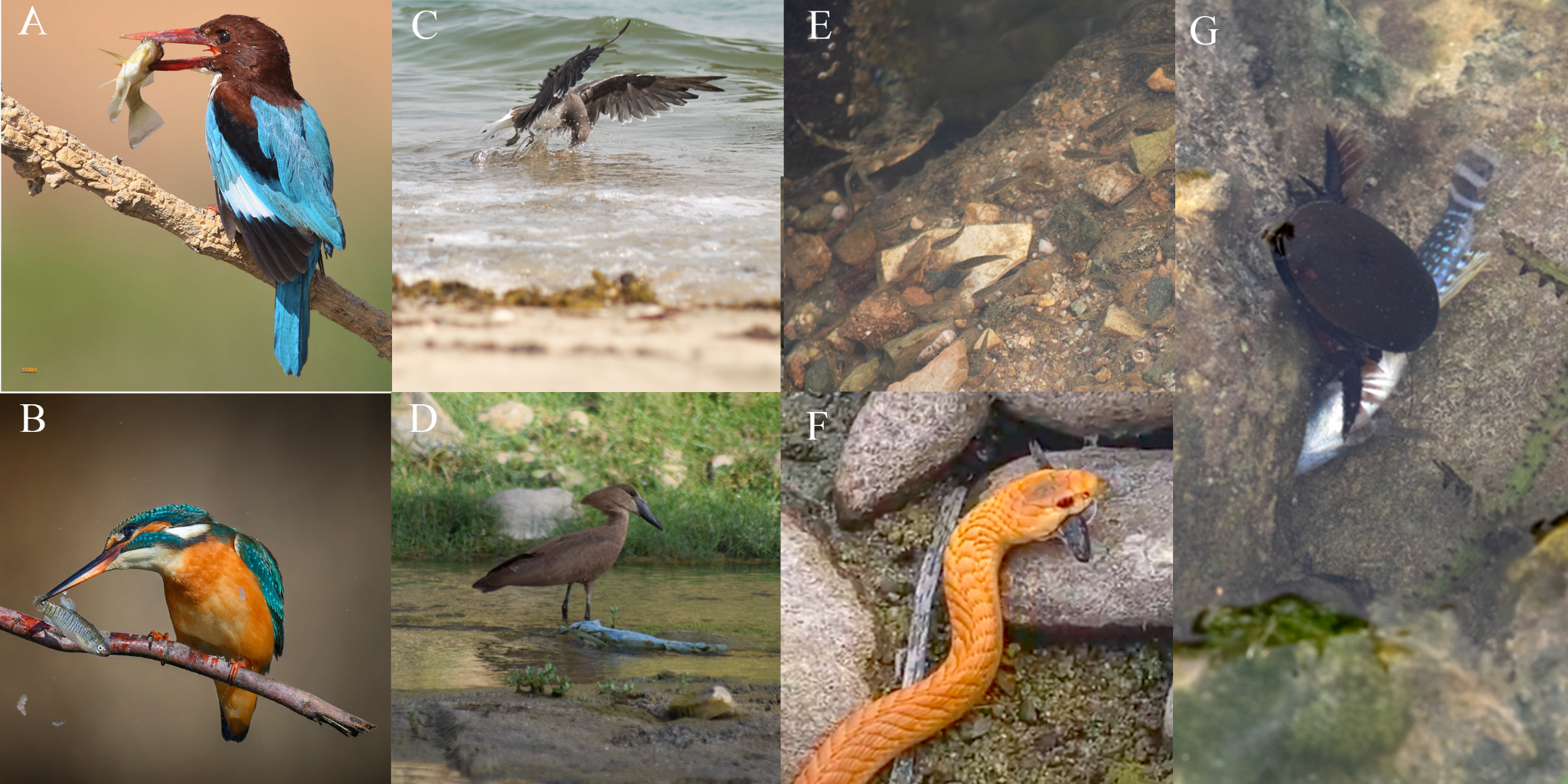


**Figure S3:** Some of the natural predators of *Aphaniops* in Saudi Arabia. (A) A white-throated kingfisher (*Halcyon smyrnensis*) feeding on a male of *A. stoliczkanus* from the Eastern province of Saudi Arabia, © Turki Alazwari. (B) A common kingfisher (*Alcedo atthis*) feeding on a female of *A. stoliczkanus* from the Eastern province of Saudi Arabia, © Ali Alhassan. (C) A sooty gull (*Ichthyaetus hemprichii*) from Dana Bay on the Gizan coast, and (D) a hamerkop (*Scopus umbretta*) from Wadi Qanuna searching in shallow water where mostly only *Aphaniops* fish live. (E) A crab and a school of *A. dispar* from Jeddah, Saudi Arabia. (F) An Arabian cobra (*Naja arabica*) feeding on the Arabian killifish’s heterospecific (*Garra tibanica*) from Wadi Al Arj, Saudi Arabia, © Naif Almalki. (G) A water beetle feeding on a male of *A. dispar* from Wadi Nawan, Saudi Arabia. The credited photos A, B and F were used with permission from the owners.

**Methods**


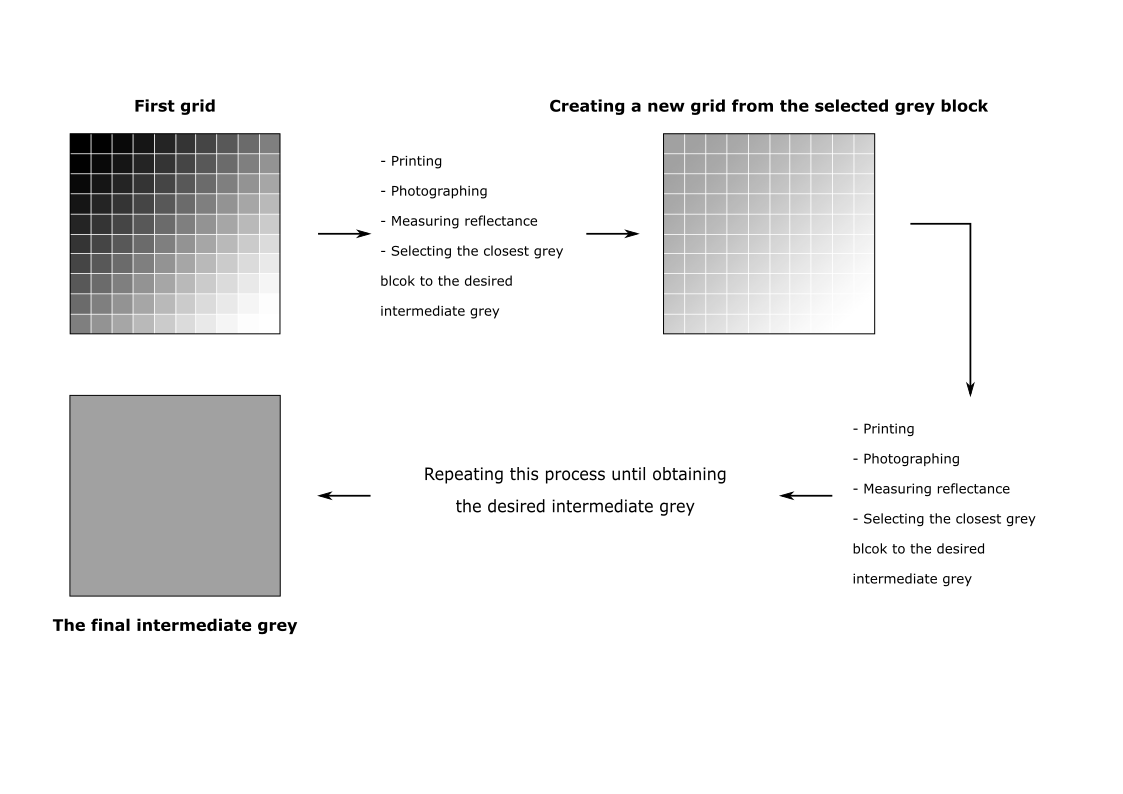


**Figure S4:** Schematic diagram illustrating the process of creating the midpoint grey colour.


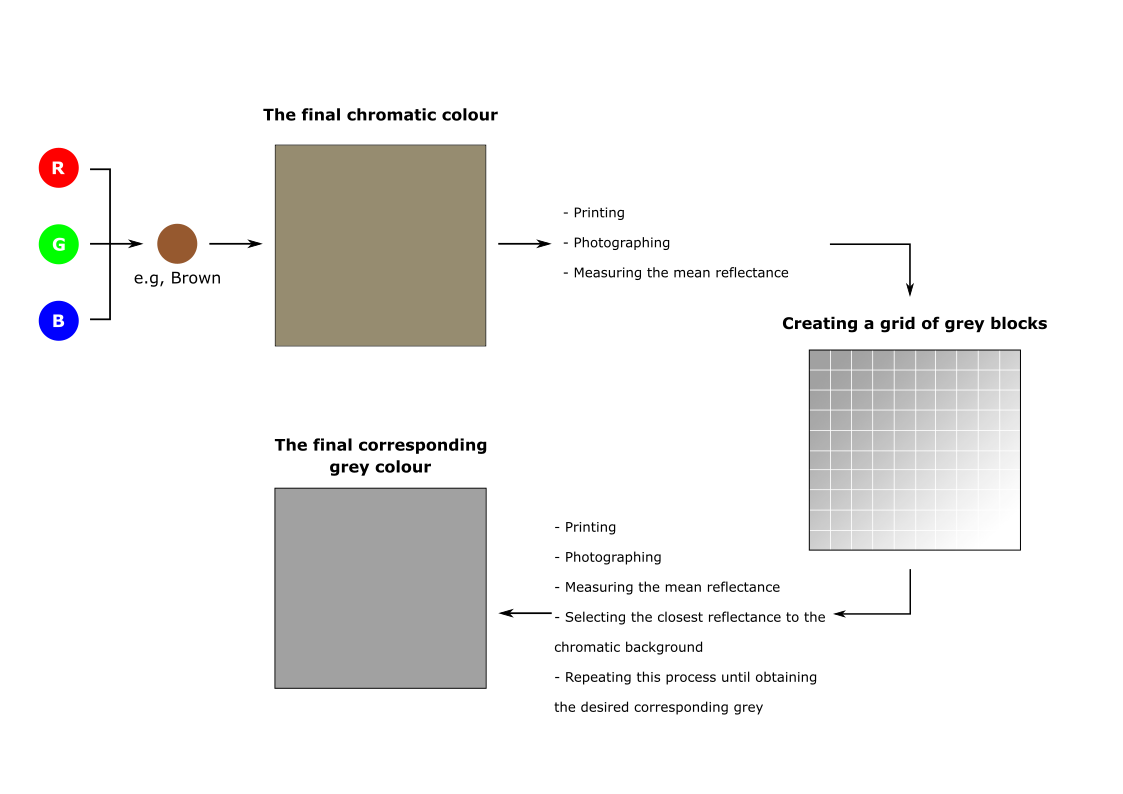


**Figure S5:** Schematic diagram illustrating an example of creating the chromatic and the corresponding achromatic backgrounds with uniform reflectance.

**Table S1:** The RGB values, mean reflectance of the beige, brown and green backgrounds used for the chromatic experiments, and the RGB of the corresponding grey value of these backgrounds as well as their mean reflectance.

| **Background colour** | **RGB values of the printed background** | **Mean reflectance of the printed background** | **RGB values of the corresponding grey background** | **Mean reflectance of the printed corresponding grey background** |
| --- | --- | --- | --- | --- |
| Beige | 169; 153; 117 | 32.4% | 132; 132; 132 | 32.38% |
| Brown | 124; 104; 070 | 18.91% | 106; 106; 106 | 18.93% |
| Green | 107; 108; 026 | 16.15% | 096; 096; 096 | 16% |

Based on the model selection approach used in the main manuscript, seven models were identified as providing a better fit than the simple model. These included the following:

***White background experiment:***

- log(LuminanceJND) ~ FishLength + FishPopulation + FishSex + TestTime + FishLength:FishSex + FishPopulation:TestTime + (1 | FishID)
- Hue ~ FishLength + FishPopulation + FishSex + TestTime + FishLength:FishSex + FishPopulation:TestTime + (1 | FishID)
- sqrt(Saturation) ~ FishLength + FishPopulation + TestTime + FishLength:TestTime + FishPopulation:TestTime + (1 | FishID)

***Beige background experiment:***

- log(Saturation) ~ FishPopulation + FishSex + TestTime + FishPopulation:FishSex + FishPopulation:TestTime + (1 | FishID)
- sqrt(ColourJND06) ~ FishPopulation + FishSex + TestTime + FishPopulation:FishSex + FishPopulation:TestTime + (1 | FishID)

***Brown background experiment:***

- Saturation ~ FishLength + FishPopulation + TestTime + FishLength:FishPopulation + FishLength:TestTime + FishPopulation:TestTime + (1 | FishID)

***Green background experiment:***

- Saturation ~ FishLength + FishPopulation + TestTime + FishLength:FishPopulation + FishPopulation:TestTime + (1 | FishID)

**Results**

**Table S2:** The statistical results of the effects of fish sex and length on the initial colour metrics in each experiment. The outputs highlighted in green indicate statistical significance. NA denotes that the variable was excluded from the final model based on the model selection approach.

| **Experiment** | **Metric** | **Fish Sex** | **Fish Length** |
| --- | --- | --- | --- |
| Black | Luminance | (*log*(LMM): *F*(1,74) = 0.1755, *p* = 0.6765) | (*log*(LMM): *F*(1,74) = 1.7926, *p* = 0.1847) |
|  | Luminance JND | (*sqrt*(LMM): *F*(1,74) = 0.5003, *p* = 0.4816) | (*sqrt*(LMM): *F*(1,74) = 0.0131, *p* = 0.9093) |
|  | Hue | (*log*(LMM): *F*(1,74) = 0.0235, *p* = 0.8785) | (*log*(LMM): *F*(1,74) = 2.5755, *p* = 0.1128) |
|  | Saturation | (*log*(LMM): *F*(1,74) = 0.1379, *p* = 0.7115) | (*log*(LMM): *F*(1,74) = 0.9595, *p* = 0.3305) |
| White | Luminance | (LMM: *F*(1,74) = 0.4703, *p* = 0.4949990) | (LMM: *F*(1,74) = 15.0176, *p* = 0.0002283) |
|  | Luminance JND | (*log*(LMM): *F*(1,73) = 6.038, *p* = 0.016) | (*log*(LMM): *F*(1,73) = 5.847, *p* = 0.018) |
|  | Hue | (LMM: *F*(1,73) = 6.579, *p* = 0.012) | (LMM: *F*(1,73) = 1.5263, *p* = 0.220624) |
|  | Saturation | NA | (sqrt(LMM): F(1,75) = 14.501, p = 0.0002) |
| Beige | Luminance | (*sqrt*(LMM): *F*(1,74) = 0.1606, *p* = 0.6898017) | (*sqrt*(LMM): *F*(1,74) = 9.7717, *p* = 0.0025323) |
|  | Luminance JND | (LMM: *F*(1,74) = 0.0307, *p* = 0.861360) | (LMM: *F*(1,74) = 8.9143, *p* = 0.003834) |
|  | Hue | (LMM: *F*(1,74) = 0.5384, *p* = 0.465398) | (LMM: *F*(1,74) = 8.1004, *p* = 0.005723) |
|  | Saturation | (*log*(LMM): *F*(1,72) = 2.3305, *p* = 0.1312) | NA |
|  | Colour JND | (sqrt(LMM): F(1,72) = 4.761, p = 0.0323) | NA |
| Brown | Luminance | (*sqrt*(LMM): *F*(1,74) = 1.5032, *p* = 0.224074) | (*sqrt*(LMM): *F*(1,74) = 2.3411, *p* = 0.130267) |
|  | Luminance JND | (LMM: *F*(1,74) = 1.9713, *p* = 0.1644889) | (LMM: *F*(1,74) = 1.7338, *p* = 0.1919922) |
|  | Hue | (*log*(LMM): *F*(1,74) = 0.4625, *p* = 0.49860) | (*log*(LMM): *F*(1,74) = 6.6960, *p* = 0.01162) |
|  | Saturation | NA | (LMM: *F*(1,72) = 0.5380, *p* = 0.465638) |
|  | Colour JND | (*log*(LMM): *F*(1,74) = 0.1494, *p* = 0.700234) | (*log*(LMM): *F*(1,74) = 3.6886, *p* = 0.058638) |
| Green | Luminance | (LMM: *F*(1,73.379) = 0.2071, *p* = 0.650415) | (LMM: *F*(1,73.351) = 4.1717, *p* = 0.044700) |
|  | Luminance JND | (*sqrt*(LMM): *F*(1,73.027) = 0.2652, *p* = 0.608109) | (*sqrt*(LMM): *F*(1,72.998) = 4.8605, *p* = 0.030630) |
|  | Hue | (*log*(LMM): *F*(1,73.387) = 0.0153, *p* = 0.9017585) | (*log*(LMM): *F*(1,73.355) = 0.0559, *p* = 0.8137584) |
|  | Saturation | NA | (LMM: *F*(1,71.505) = 0.0654, *p* = 0.798907) |
|  | Colour JND | (LMM: *F*(1,73.935) = 1.2529, *p* = 0.2666292) | (LMM: *F*(1,73.919) = 2.7314, *p* = 0.1026345) |


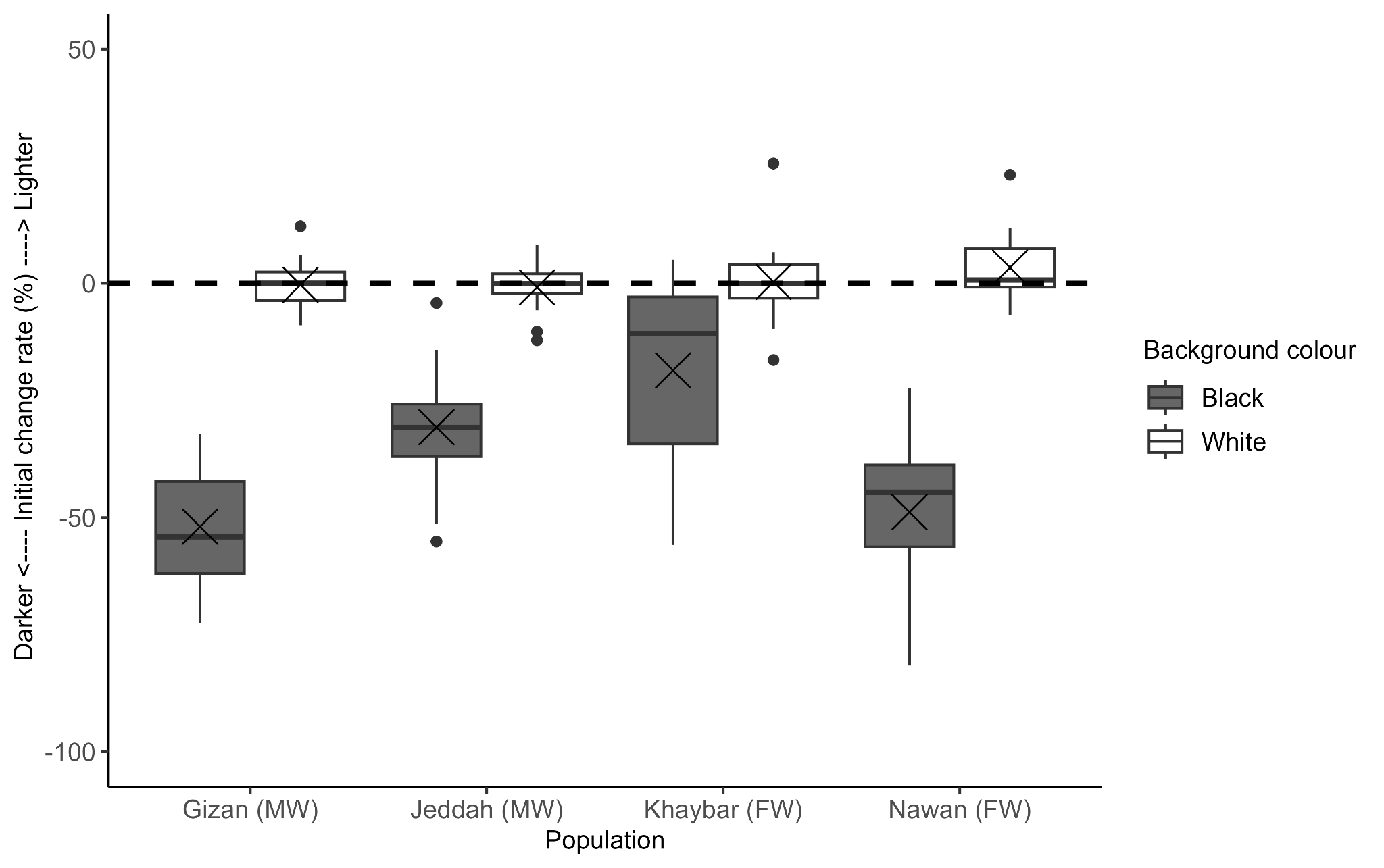


**Figure S6:** Differences among populations in the initial relative change rate [$((Minute 01 - Minute 00) \div Minute 00) \times100$] in the achromatic black and white background experiments. Boxes show means (black crosses), medians (black lines) and interquartile ranges, whiskers represent the lowest and highest values within 1.5 of the interquartile ranges, and dots represent outliers. The horizontal black dashed line represents the starting point (0 %) where positive changes indicate lightning responses and negative changes indicate darkening responses. Following the population names, the letters in parenthesis FW and MW denote the freshwater and marine habitat types, respectively.


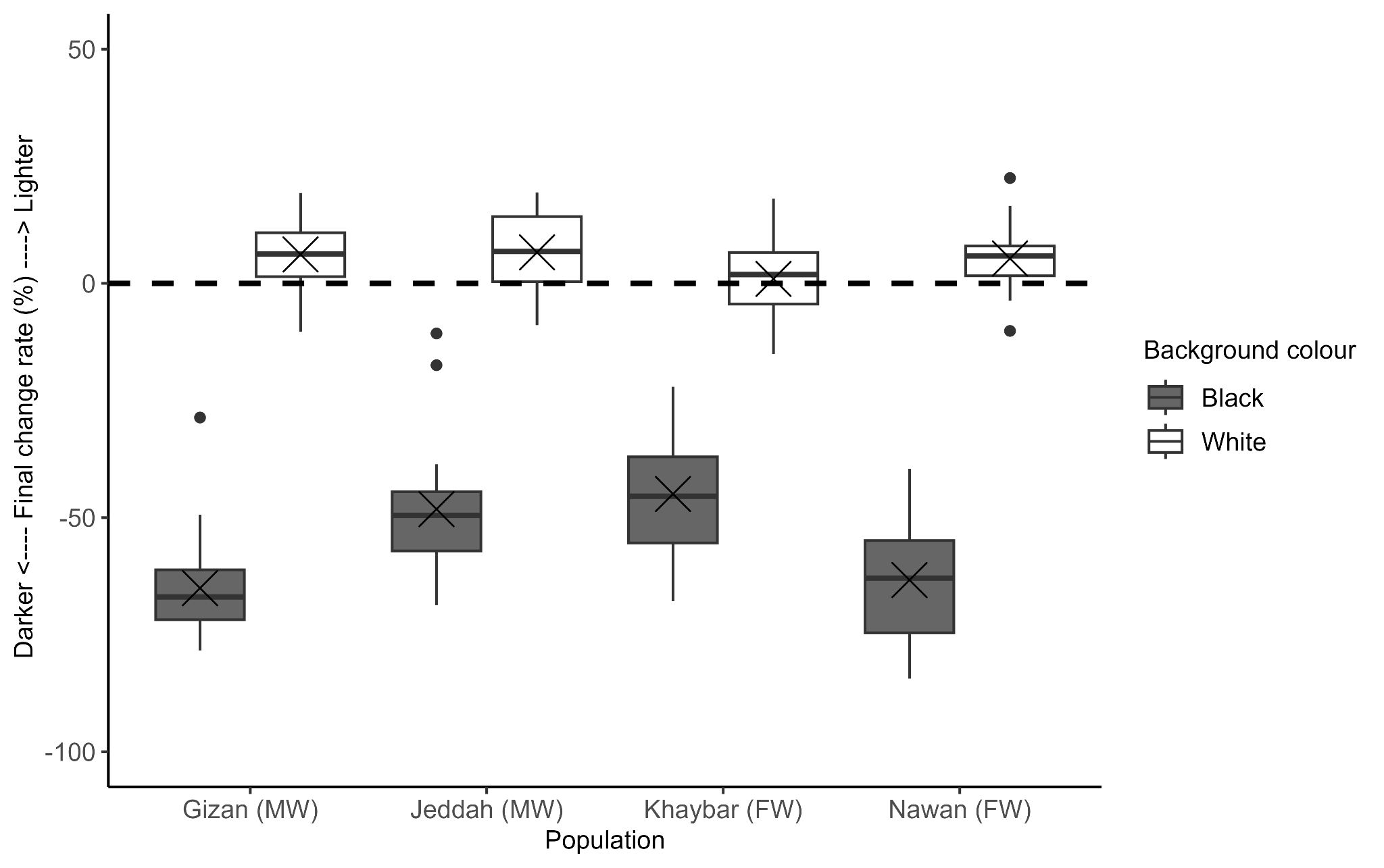


**Figure S7:** Differences among populations in the final relative change rate [$((Minute 15 - Minute 00) \div Minute 00) \times100$] in the achromatic black and white background experiments. Boxes show means (black crosses), medians (black lines) and interquartile ranges, whiskers represent the lowest and highest values within 1.5 of the interquartile ranges, and dots represent outliers. The horizontal black dashed line represents the starting point (0 %) where positive changes indicate lightning responses and negative changes indicate darkening responses. Following the population names, the letters in parenthesis FW and MW denote the freshwater and marine habitat types, respectively.


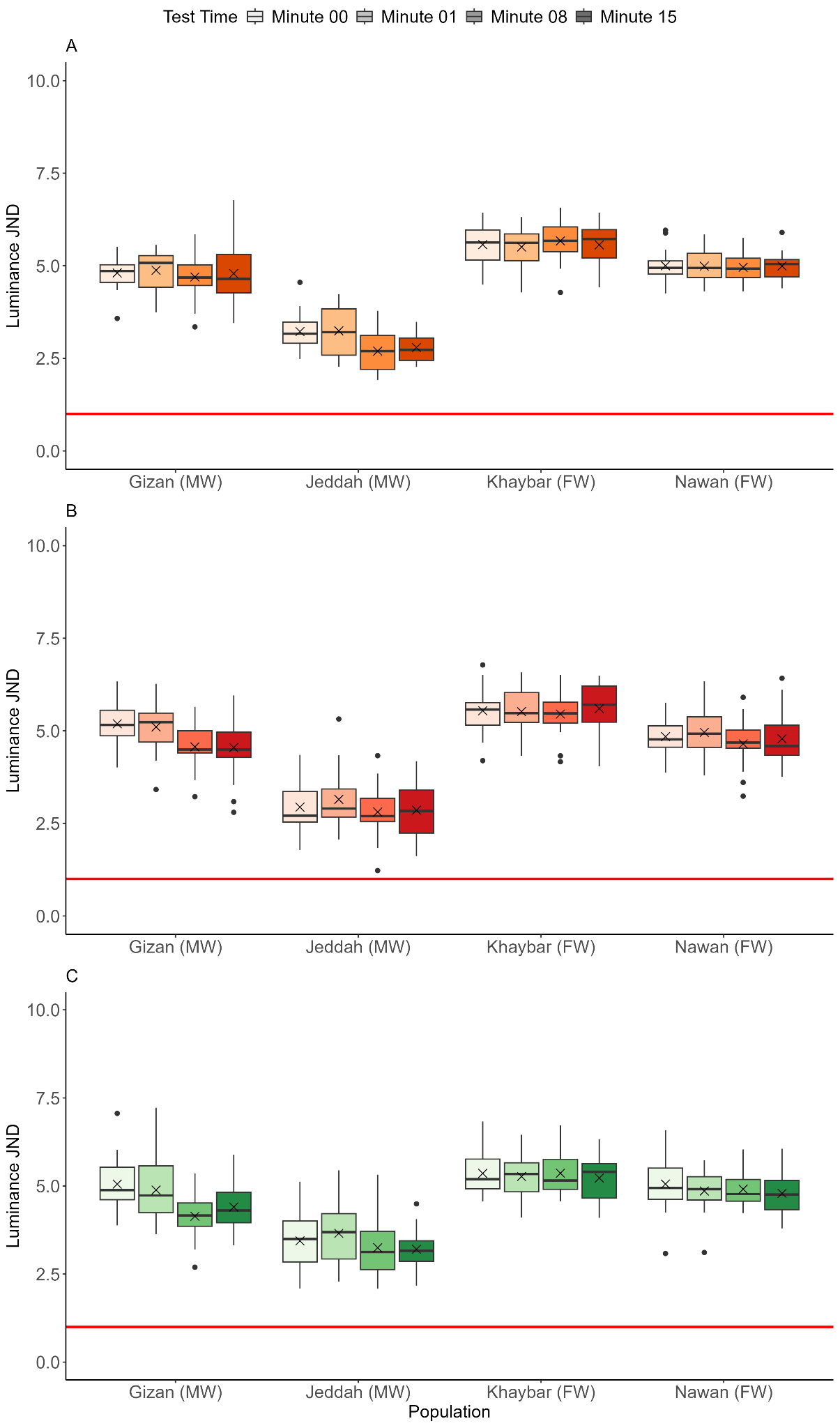


**Figure S8:** Changes in the luminance JND within 15 minutes in (A) the beige, (B) brown, and (C) green experiments (*n* = 20 per population; only the Nawan population had 19 individuals in the green background experiment at minute 08). The boxes show the means (black crosses), medians (black lines) and interquartile ranges. The whiskers represent the lowest and highest values within 1.5 × the interquartile ranges, and the dots represent the outliers. The horizontal solid red line represents the detection threshold of luminance contrast at 1 JND by the avian model. The light-to-dark coloured guides correspond to the test time. Following the population names, the letters in parenthesis FW and MW denote the freshwater and marine habitat types, respectively.
